# Supplementary material for: The Reactions of Alkenes with Phenyl-N-triflylimino-λ3-iodane: Solvent and Oxidant Impact
Source: Int J Mol Sci. 2023 Nov 3;24(21):15947. doi: 10.3390/ijms242115947 (PMC10650846; doi:10.3390/ijms242115947)

Supplementary Information:

The Reactions of Alkenes with Phenyl-*N*-triflylimino-λ^3-iodane: Solvent and Oxidant Impact

Mikhail Yu. Moskalik , Anton S. Ganin * and Bagrat A. Shainyan

A.E. Favorsky Irkutsk Institute of Chemistry, Siberian Division of the Russian Academy of Sciences, 1 Favorsky Street, 664033 Irkutsk, Russia; moskalik@irioch.irk.ru (M.Y.M.); bagrat@irioch.irk.ru (B.A.S.)
* Correspondence: ganin@irioch.irk.ru

Table of Contents

1.1 Copies of NMR Spectra S1
1.2 Copies of HRMS Spectra S40

1.1 Copies of NMR spectra

Figure S1. ^{1}H NMR (400.1 MHz, $CDCl_3$) of compound **3**

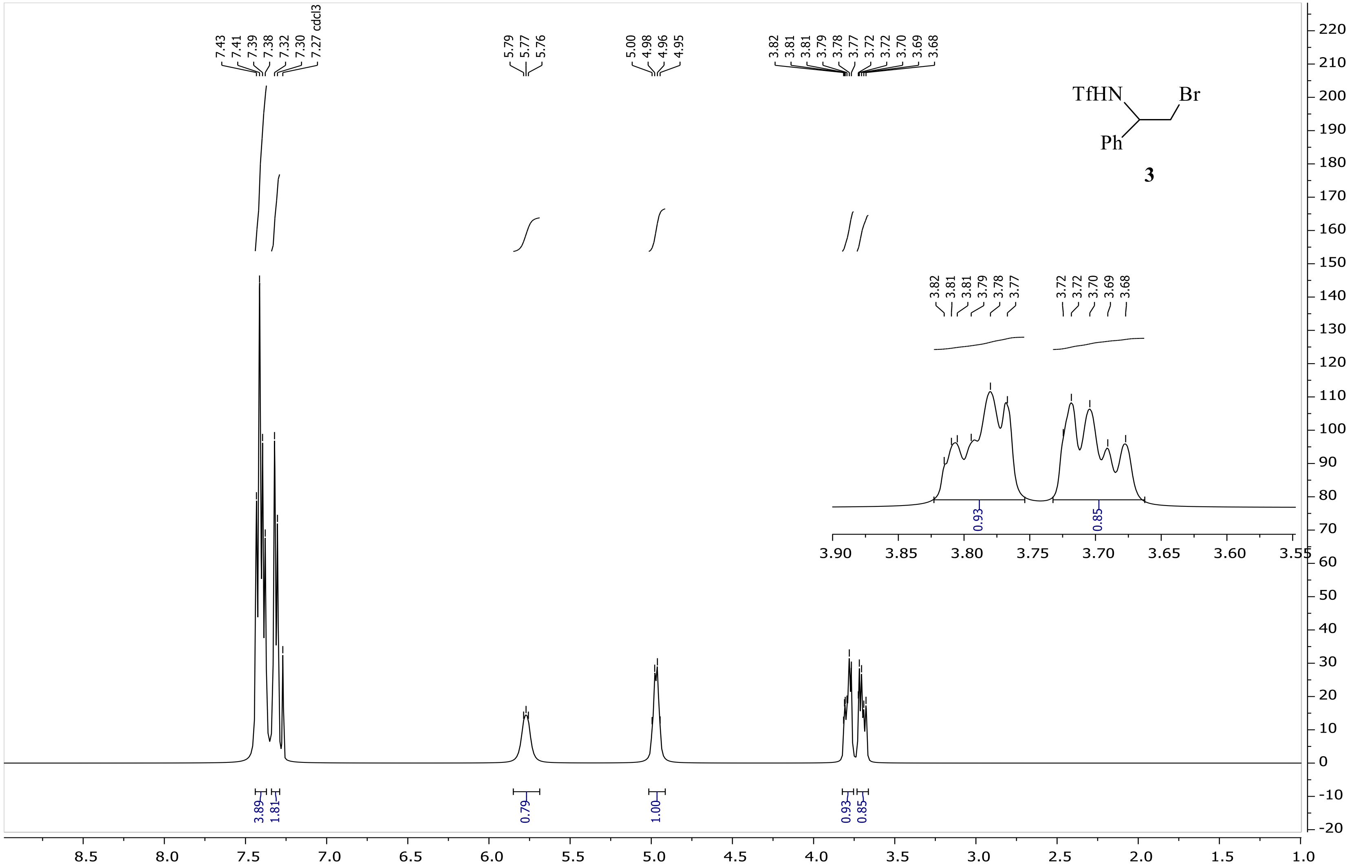

Figure S2. ^{13}C NMR (100.6 MHz, $CDCl_3$) of compound **3**

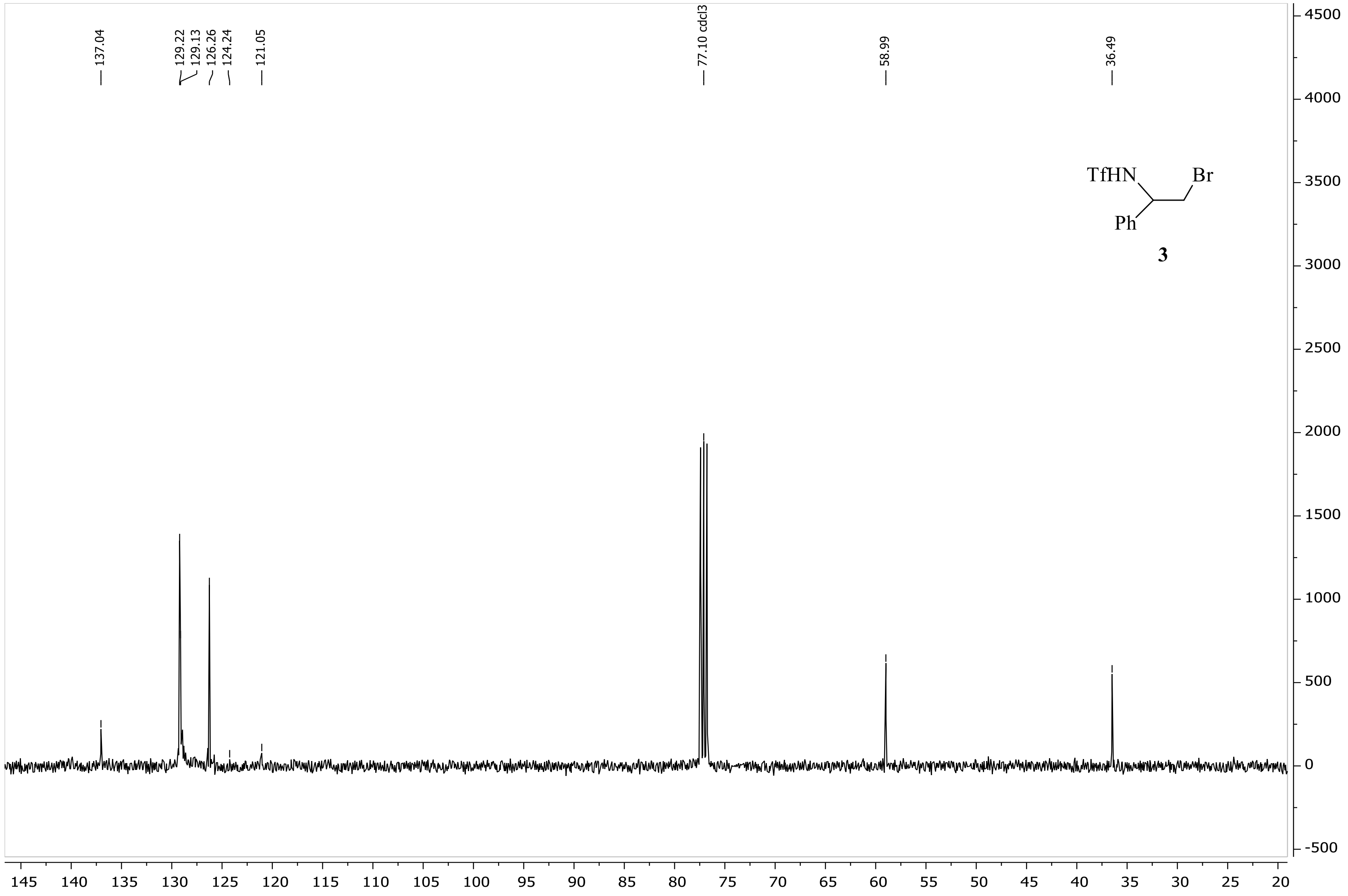

Figure S3. ^{19}F NMR (376 MHz) of compound **3**

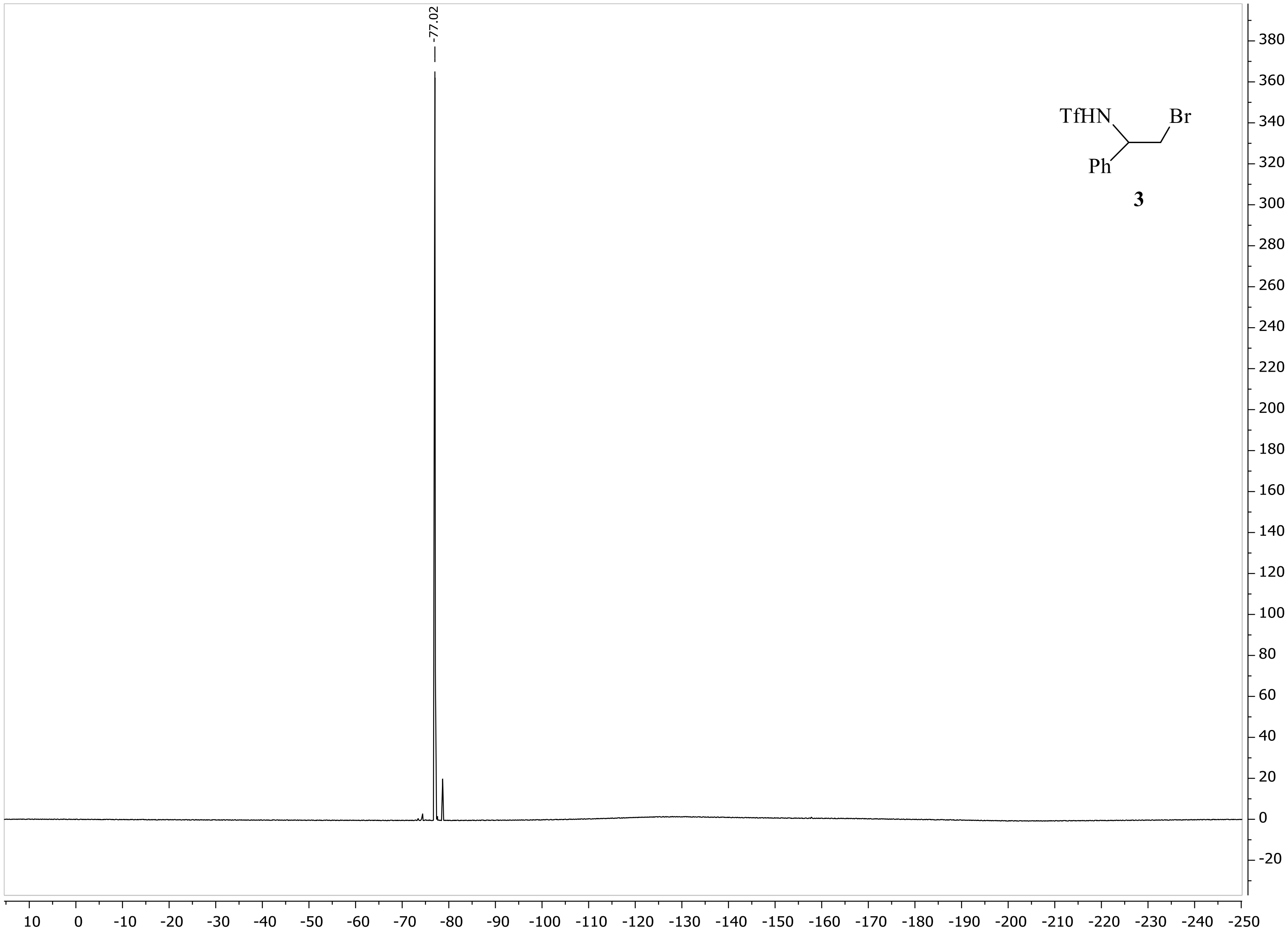

Figure S4. ^{1}H NMR (400.1 MHz, $CDCl_3$) of compound **4**

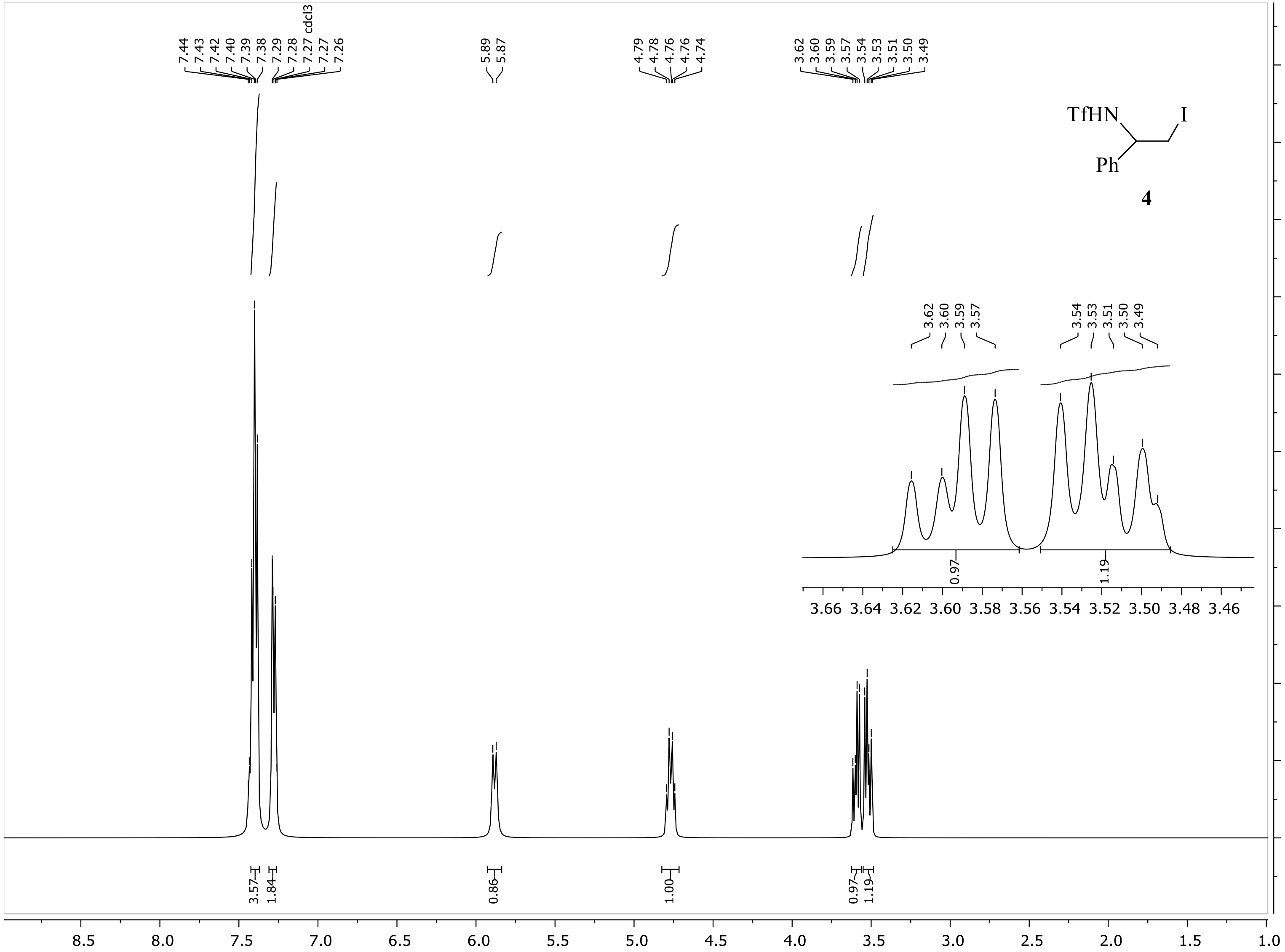

Figure S5. ^{13}C NMR (100.6 MHz, $CDCl_3$) of compound **4**

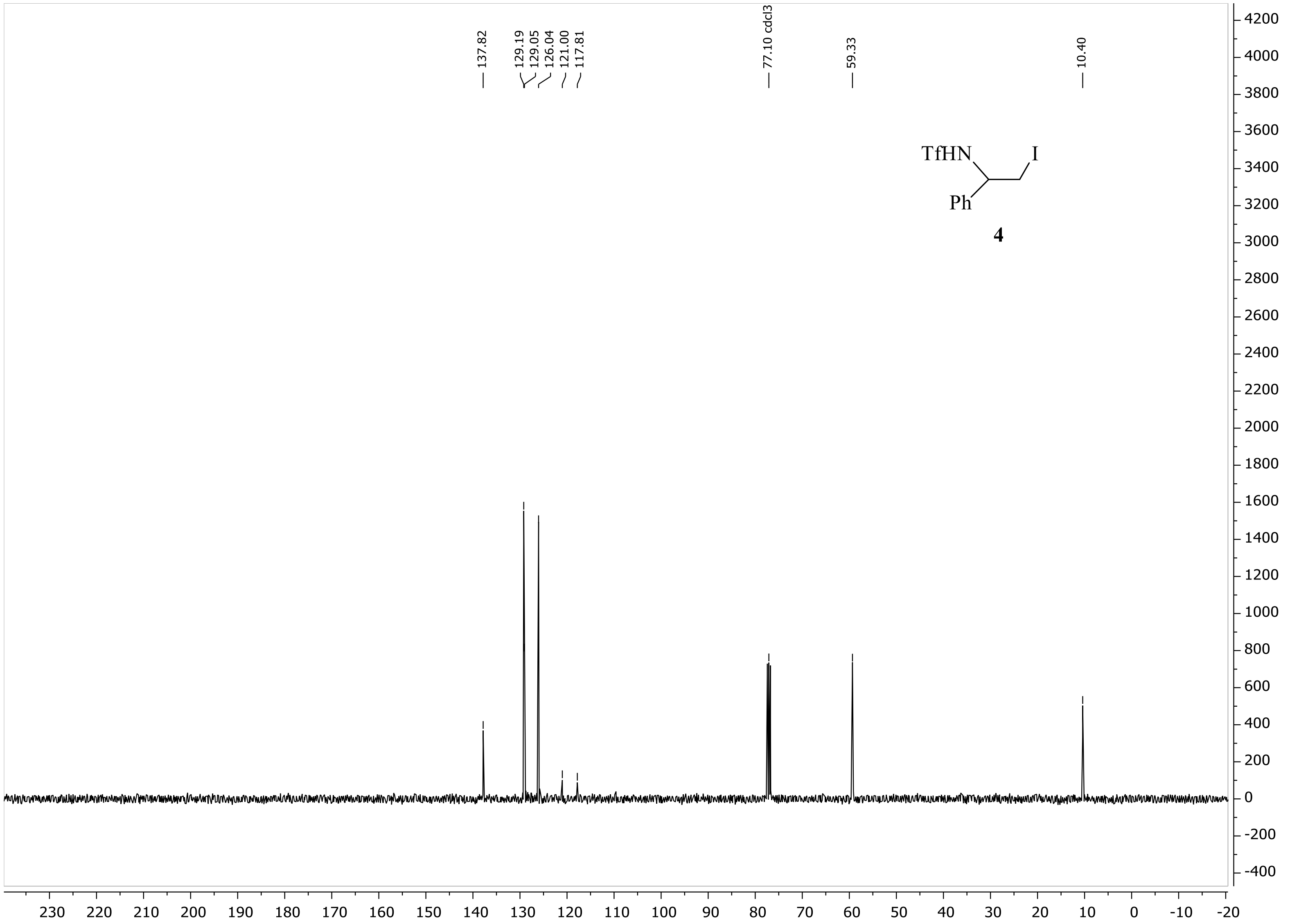

Figure S6. ^{19}F NMR (376 MHz) of compound **4**

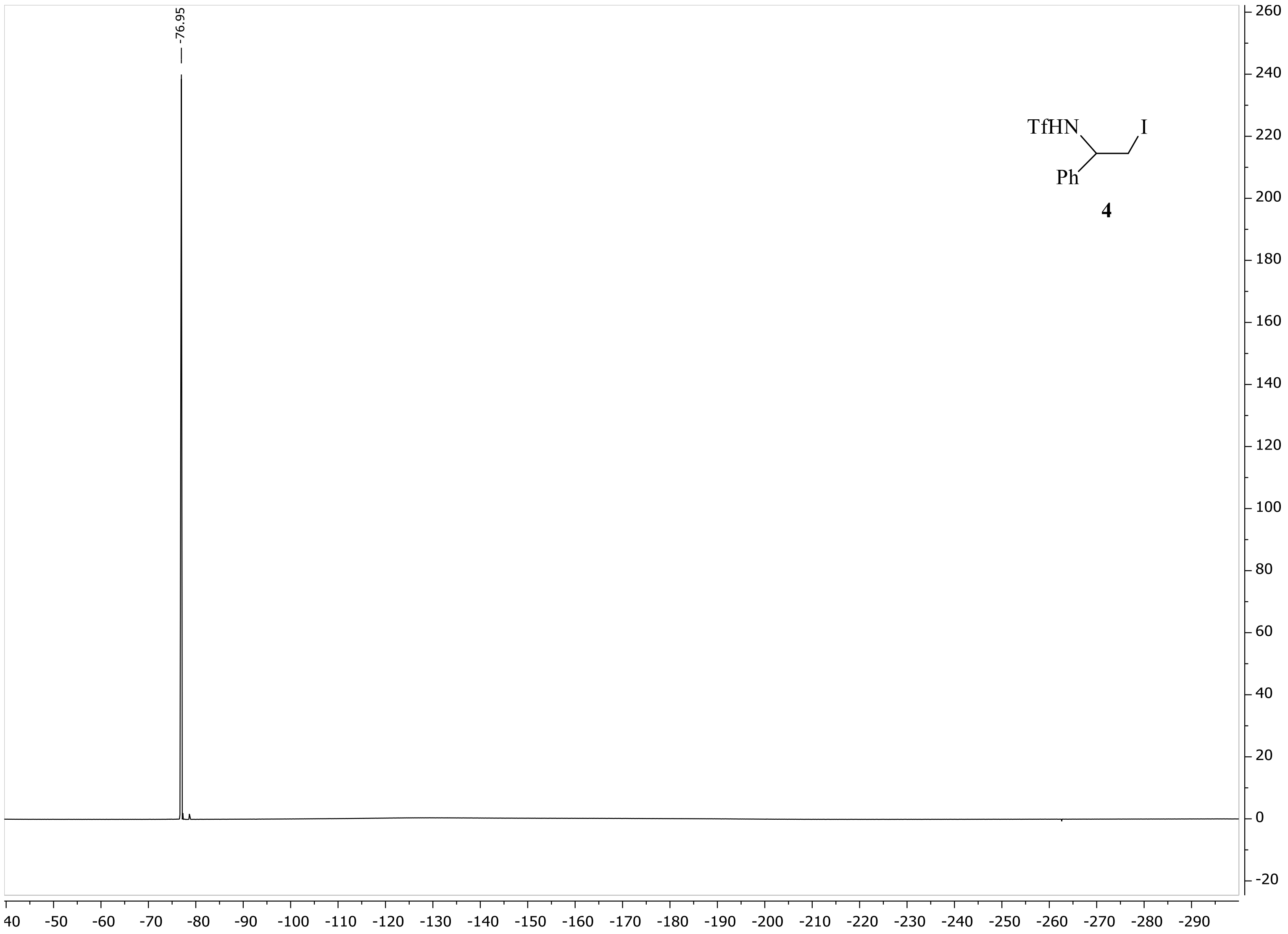

Figure S7. ^{1}H NMR (400.1 MHz, $CDCl_3$) of compound **6**

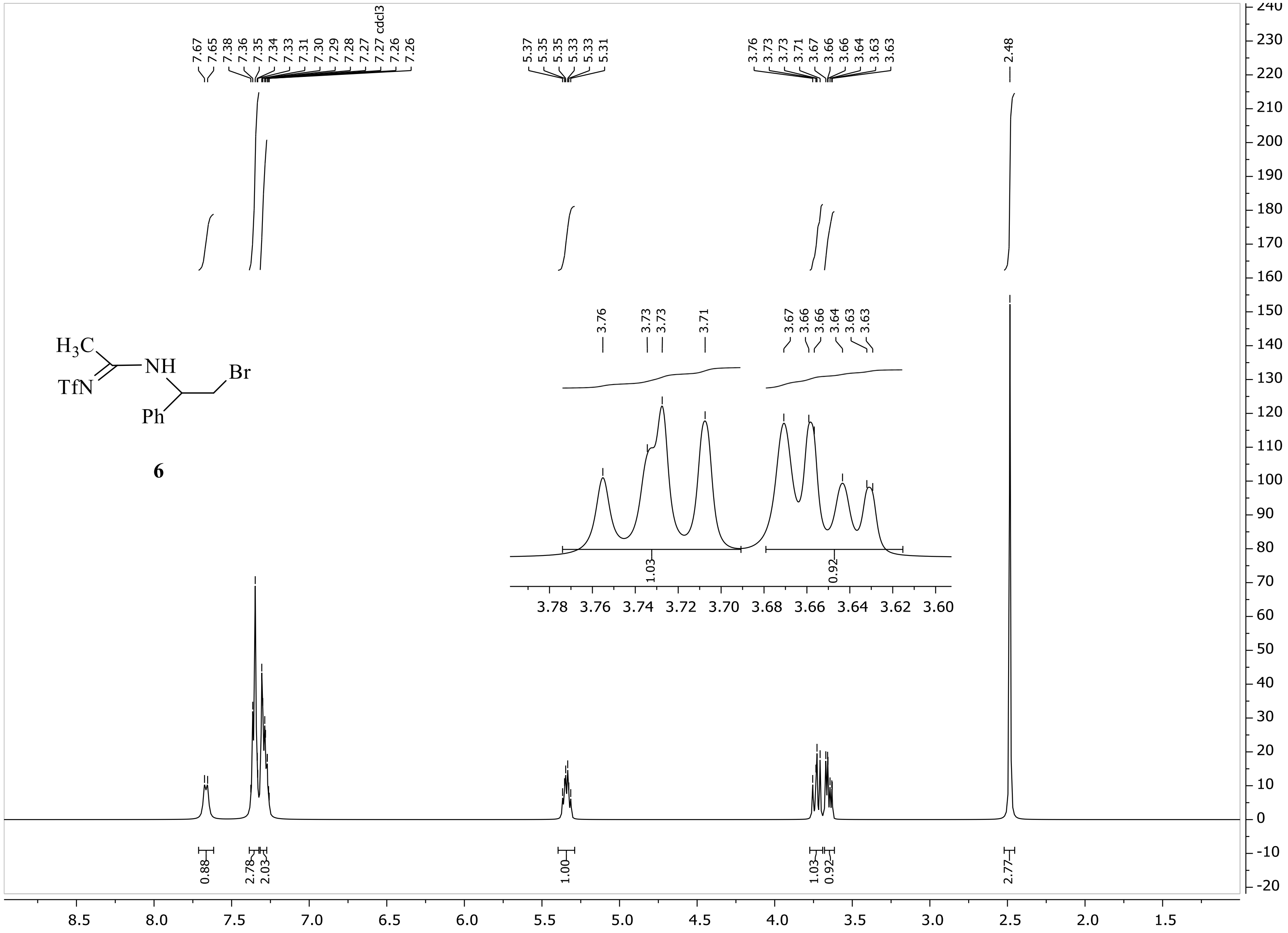

6

Figure S8. ^{13}C NMR (100.6 MHz, $CDCl_3$) of compound **6**

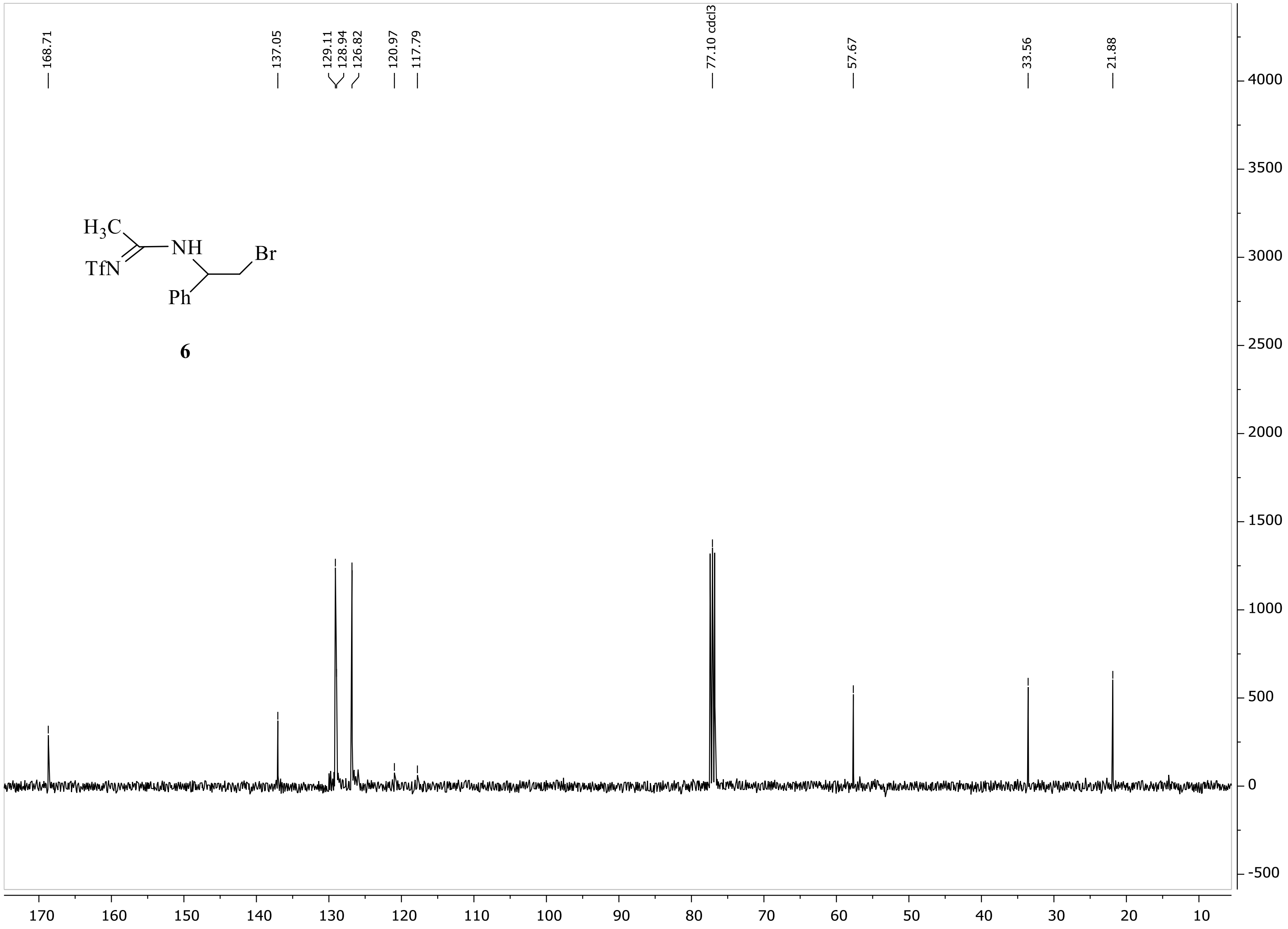

Figure S9. ^{19}F NMR (376 MHz) of compound **6**

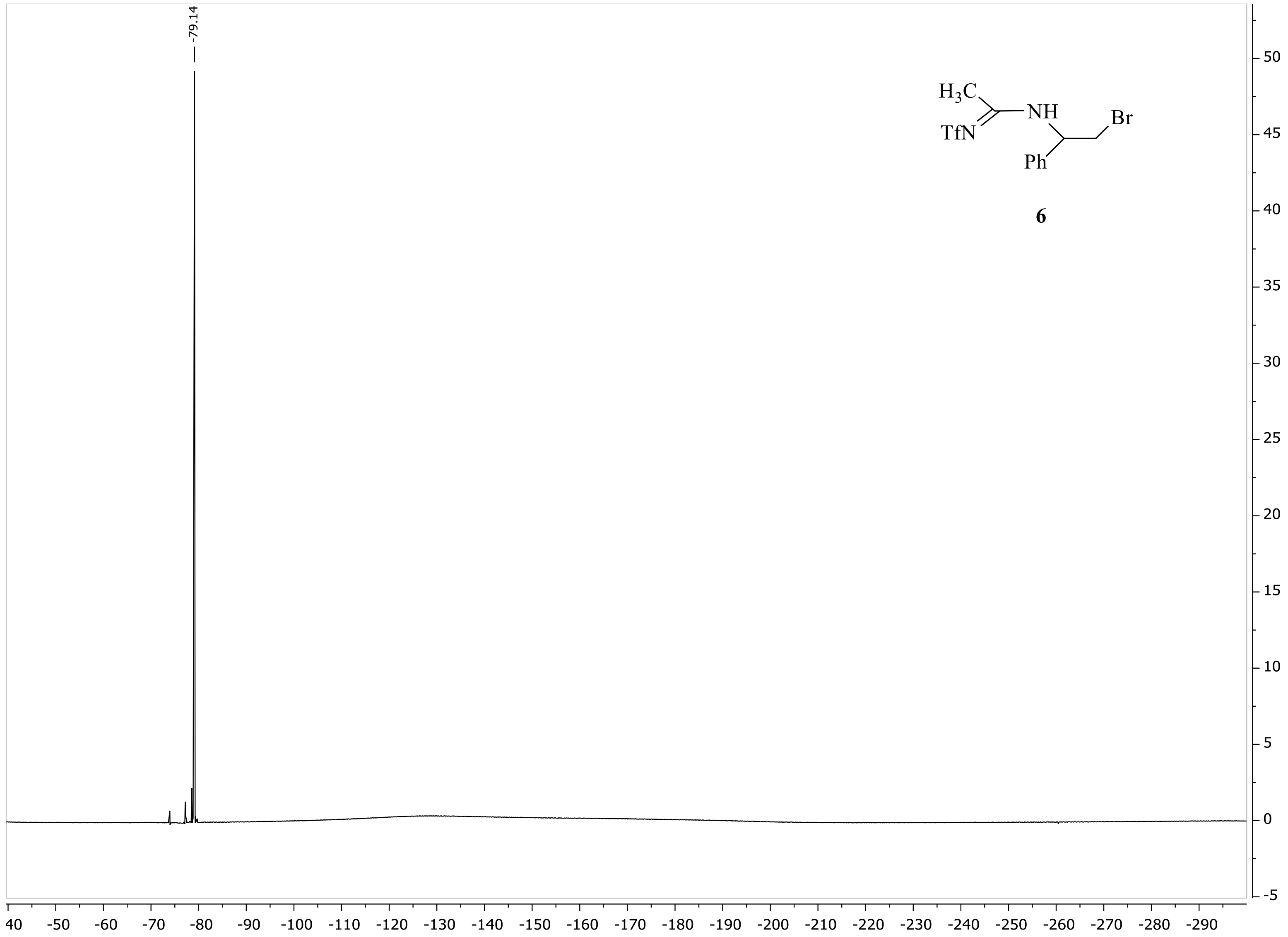

Figure S10. ^{1}H NMR (400.1 MHz, $CDCl_3$) of compound **9**

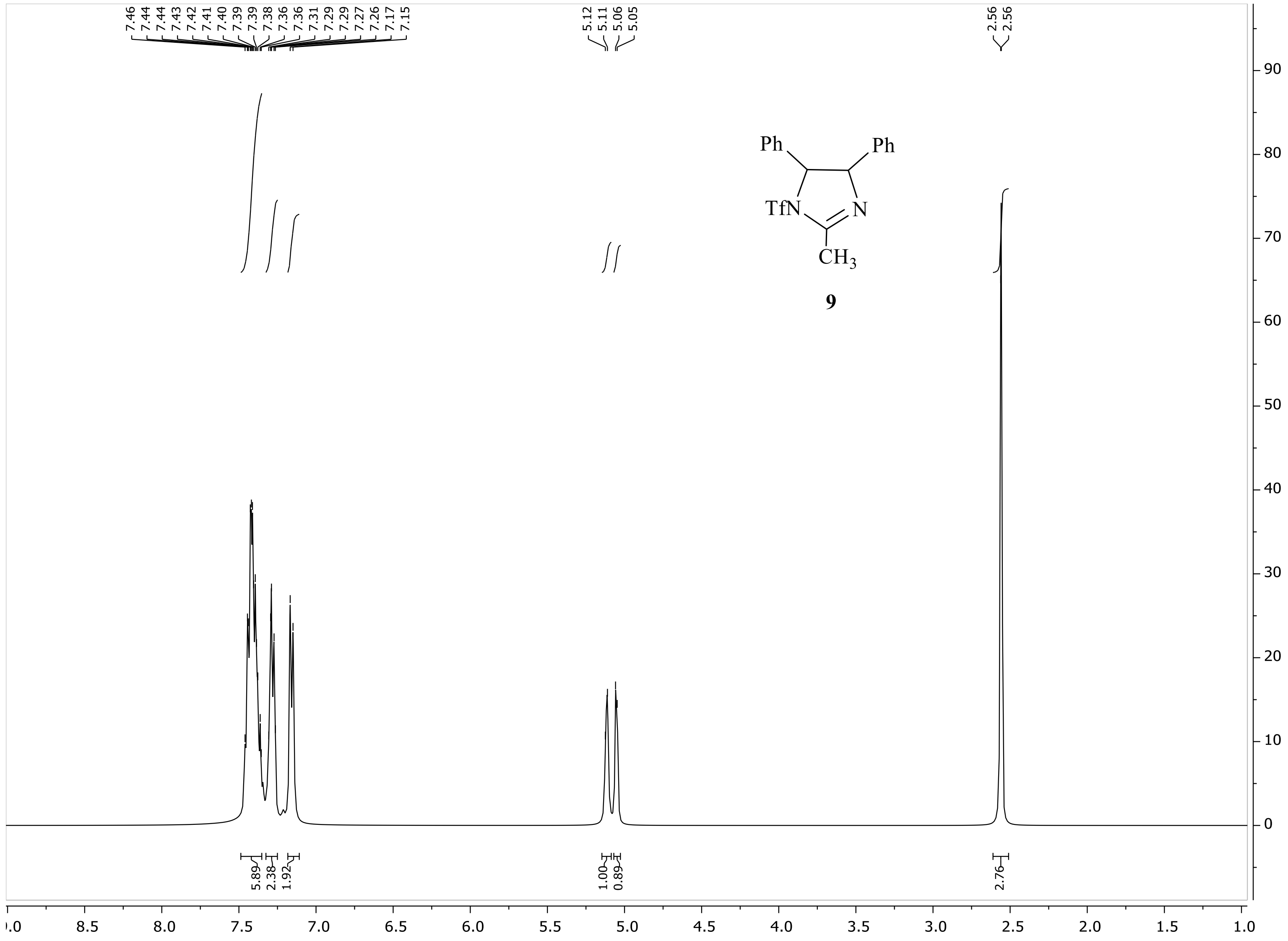

Figure S11. ^{13}C NMR (100.6 MHz, $CDCl_3$) of compound **9**

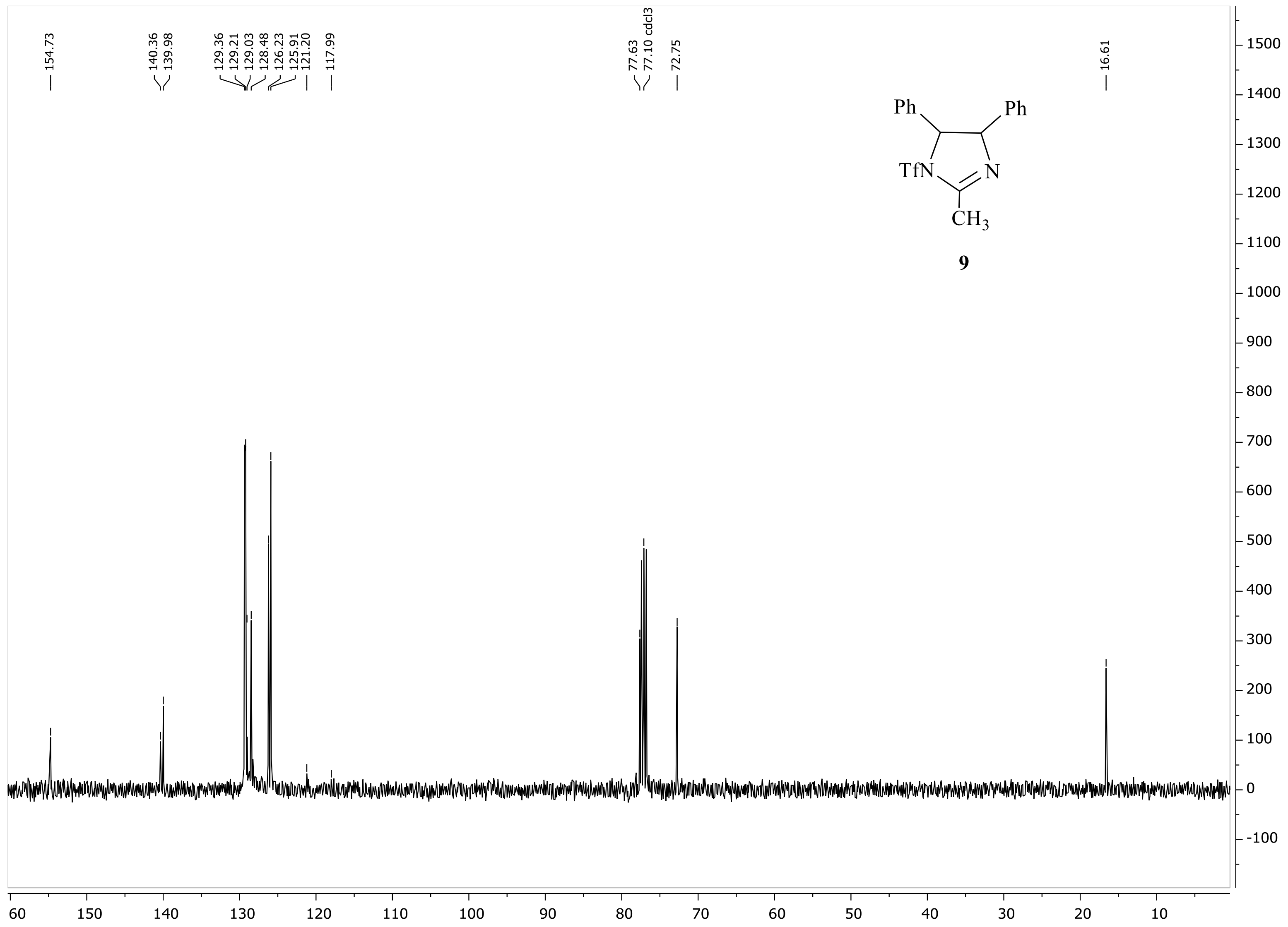

Figure S12. ^{19}F NMR (376 MHz) of compound **9**

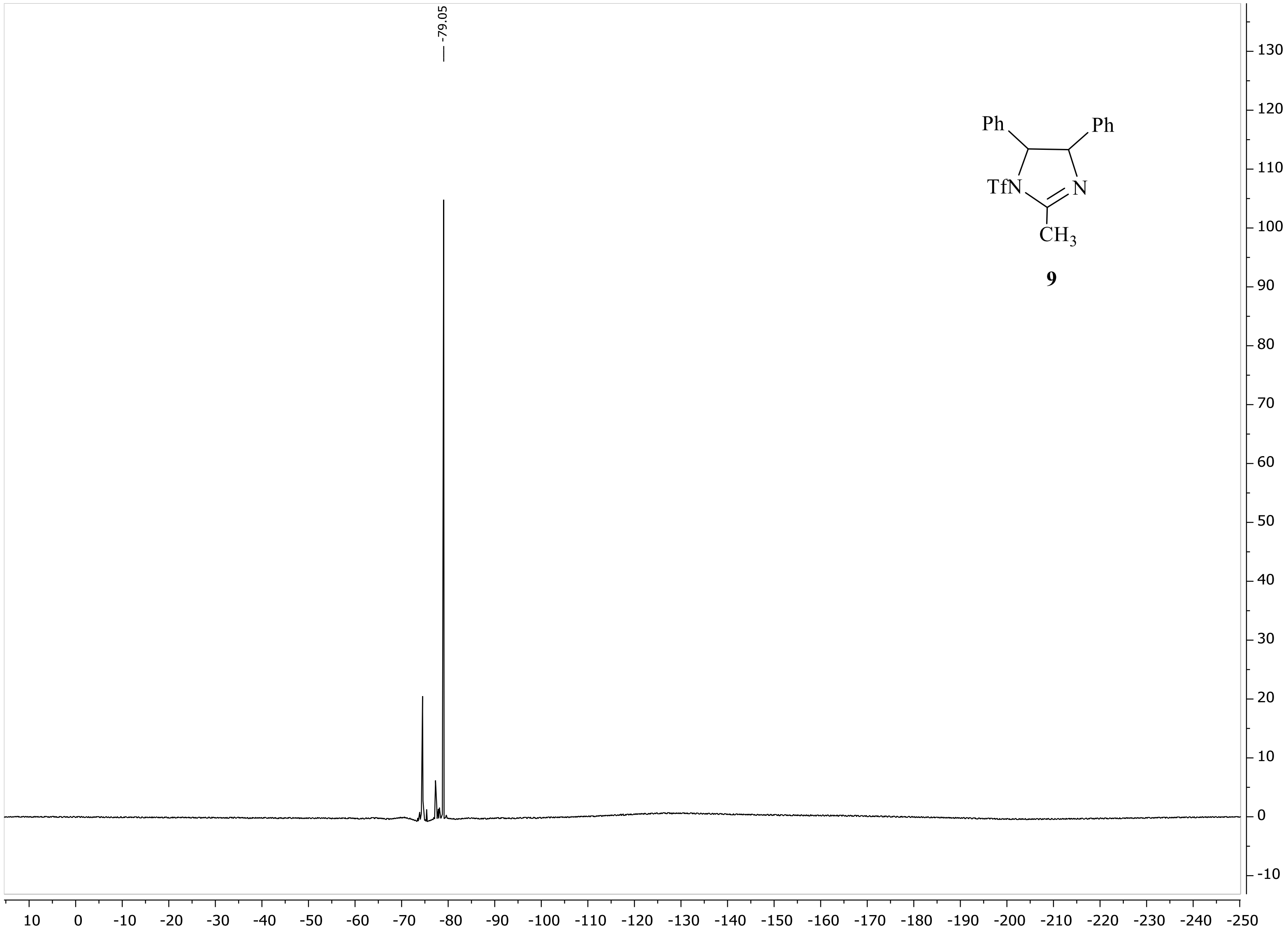

Figure S13. ^{1}H NMR (400.1 MHz, $CDCl_3$) of compound **10**

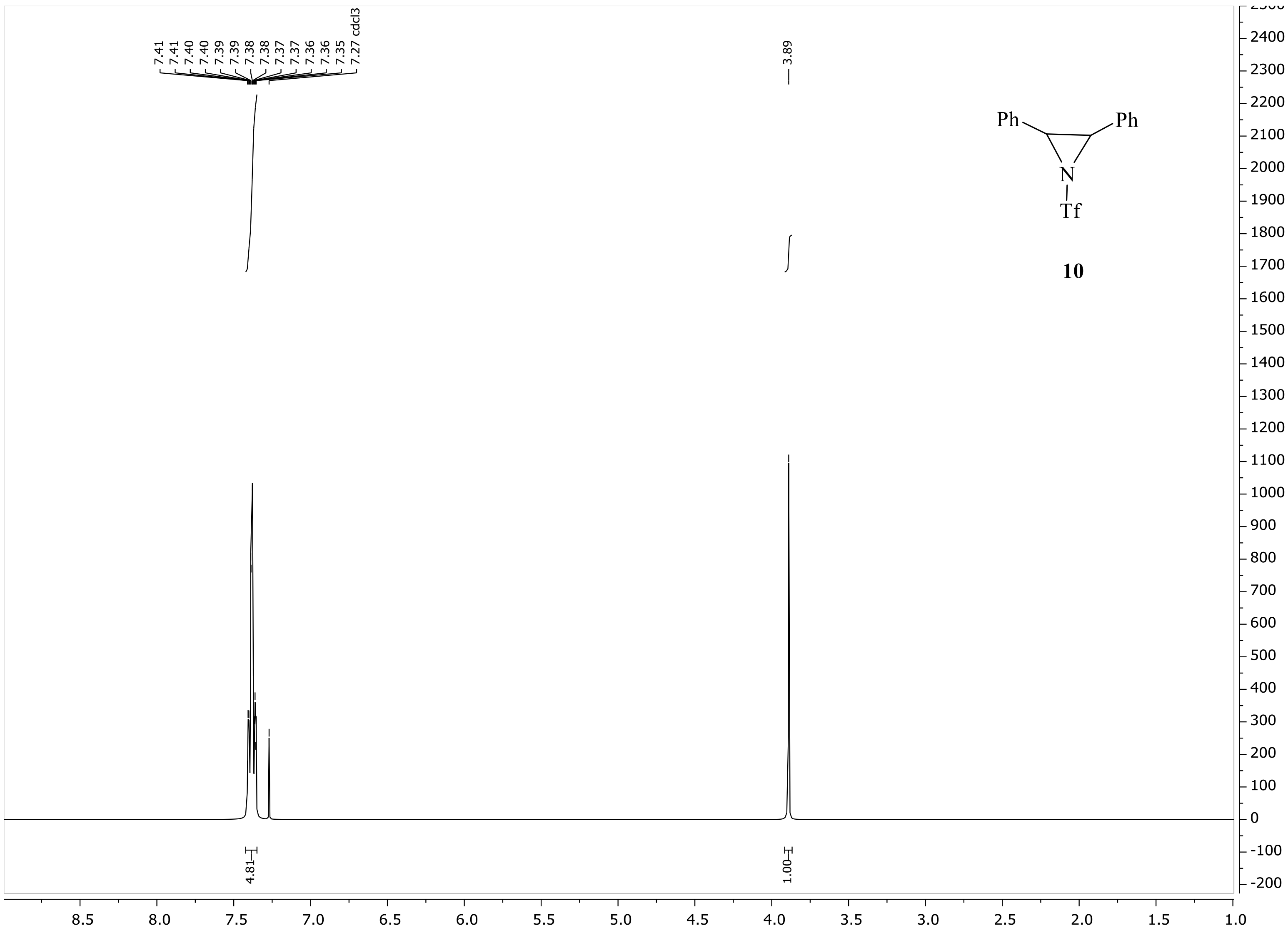

Figure S14. ^{13}C NMR (100.6 MHz, CDCl$_3$) of compound **10**

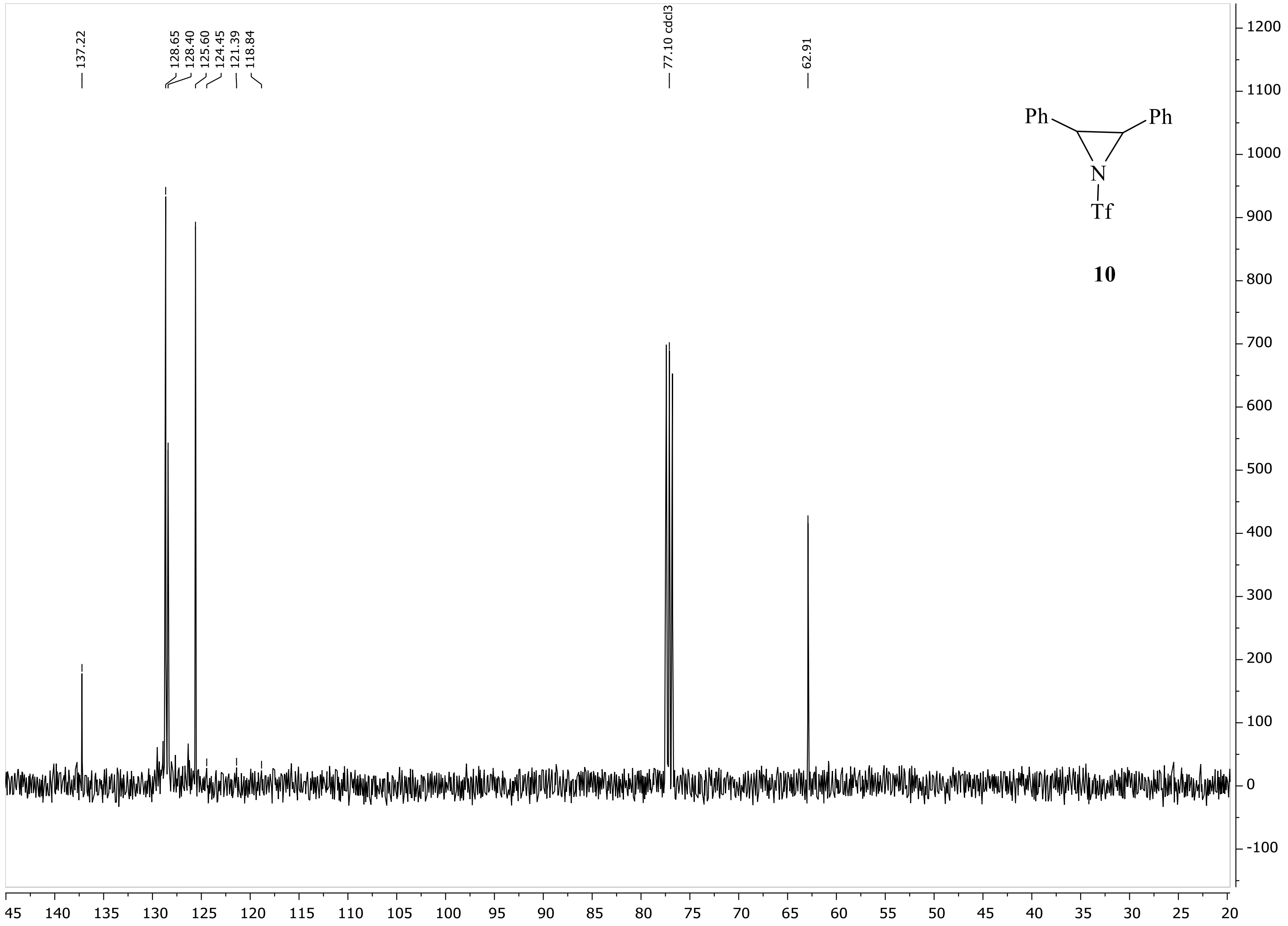

Figure S15. ^{13}C ([^{13}C – ^{1}H]) NMR (100.6 MHz, $CDCl_3$) of compound **10**

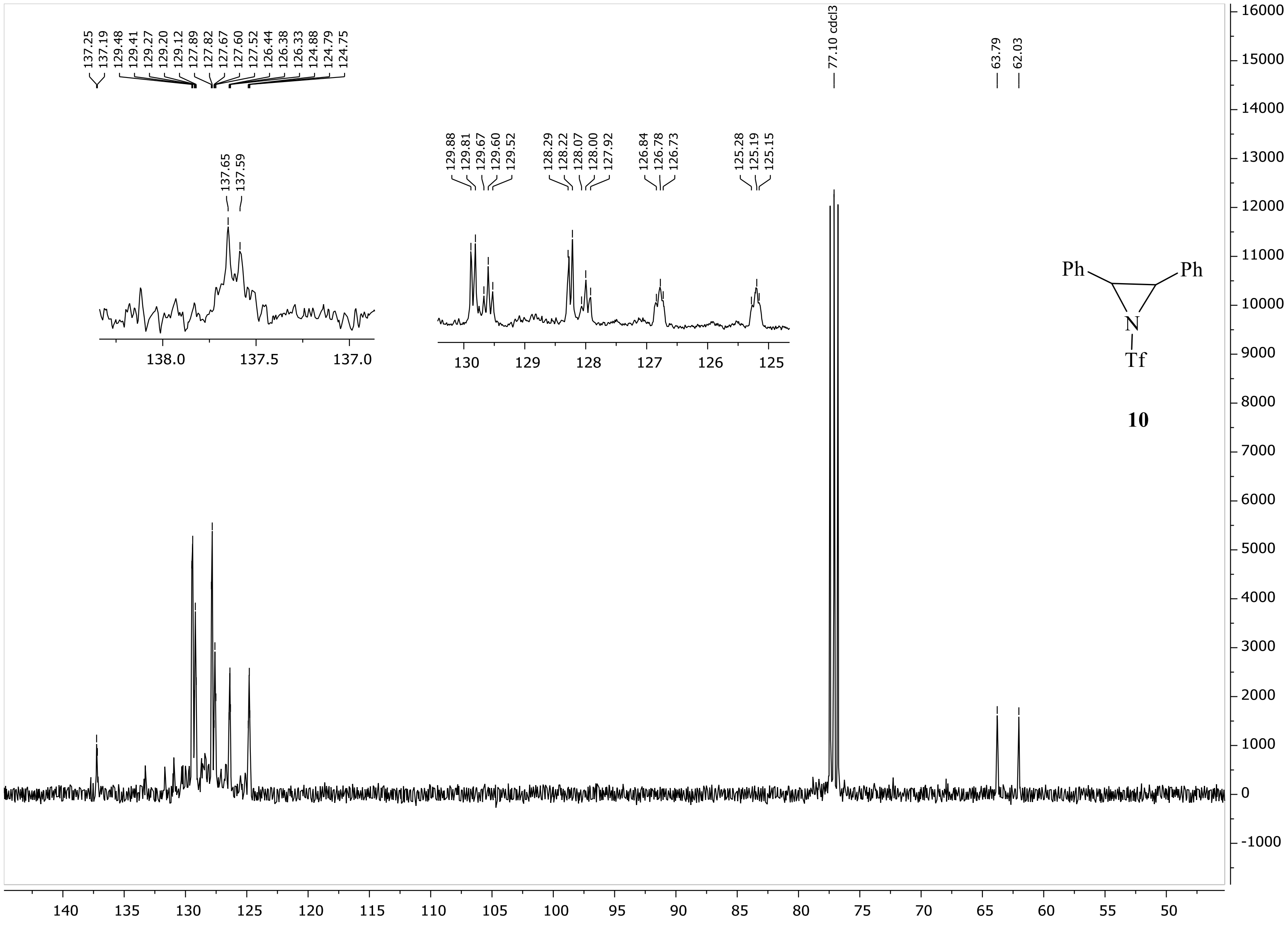

Figure S16. ^{19}F NMR (376 MHz) of compound **10**

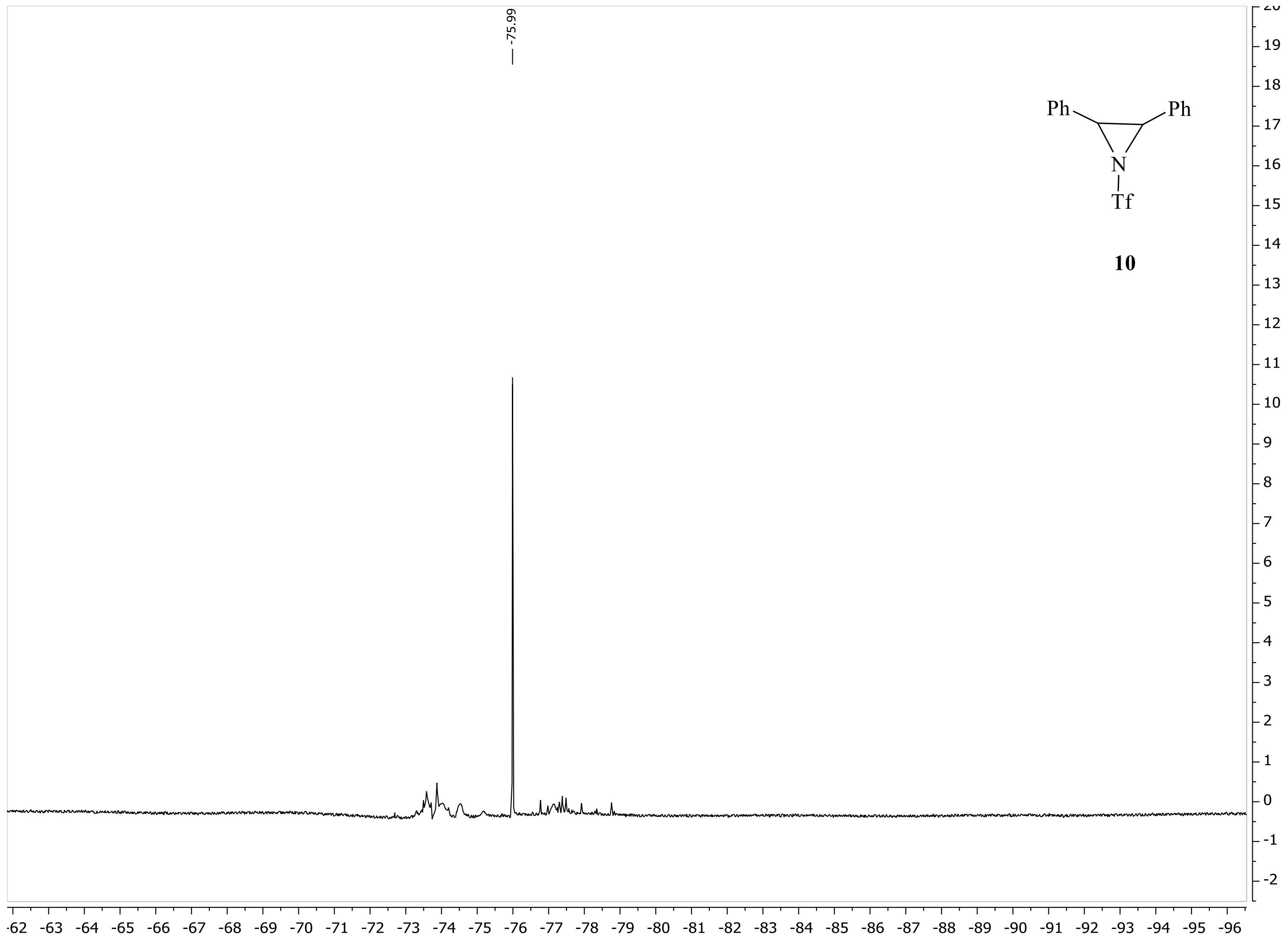

Figure S17. ^{1}H NMR (400.1 MHz, $CDCl_3$) of compound **11** (pure)

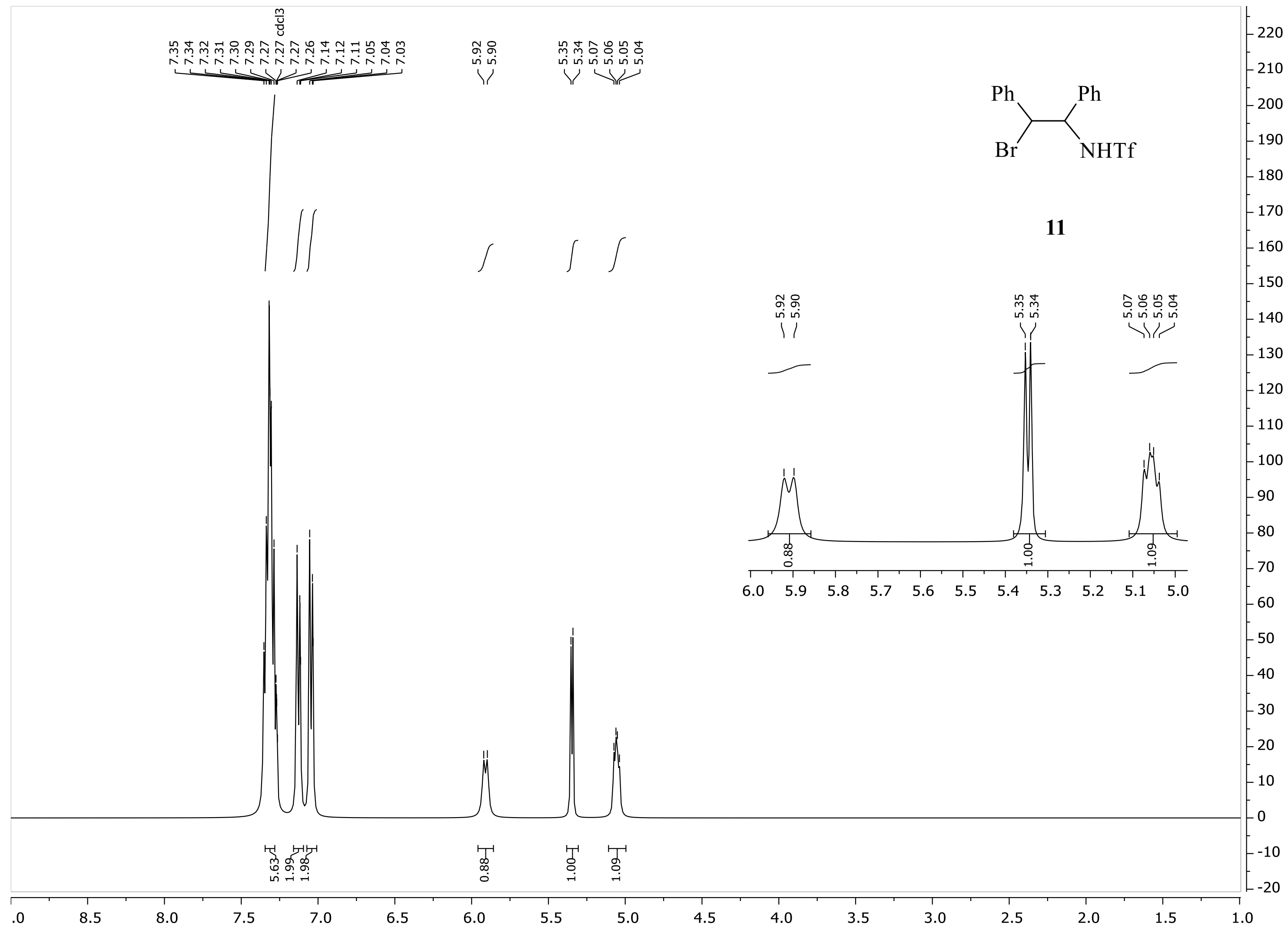

Figure S18. ^{1}H NMR (400.1 MHz, $CDCl_3$) of compound **11**

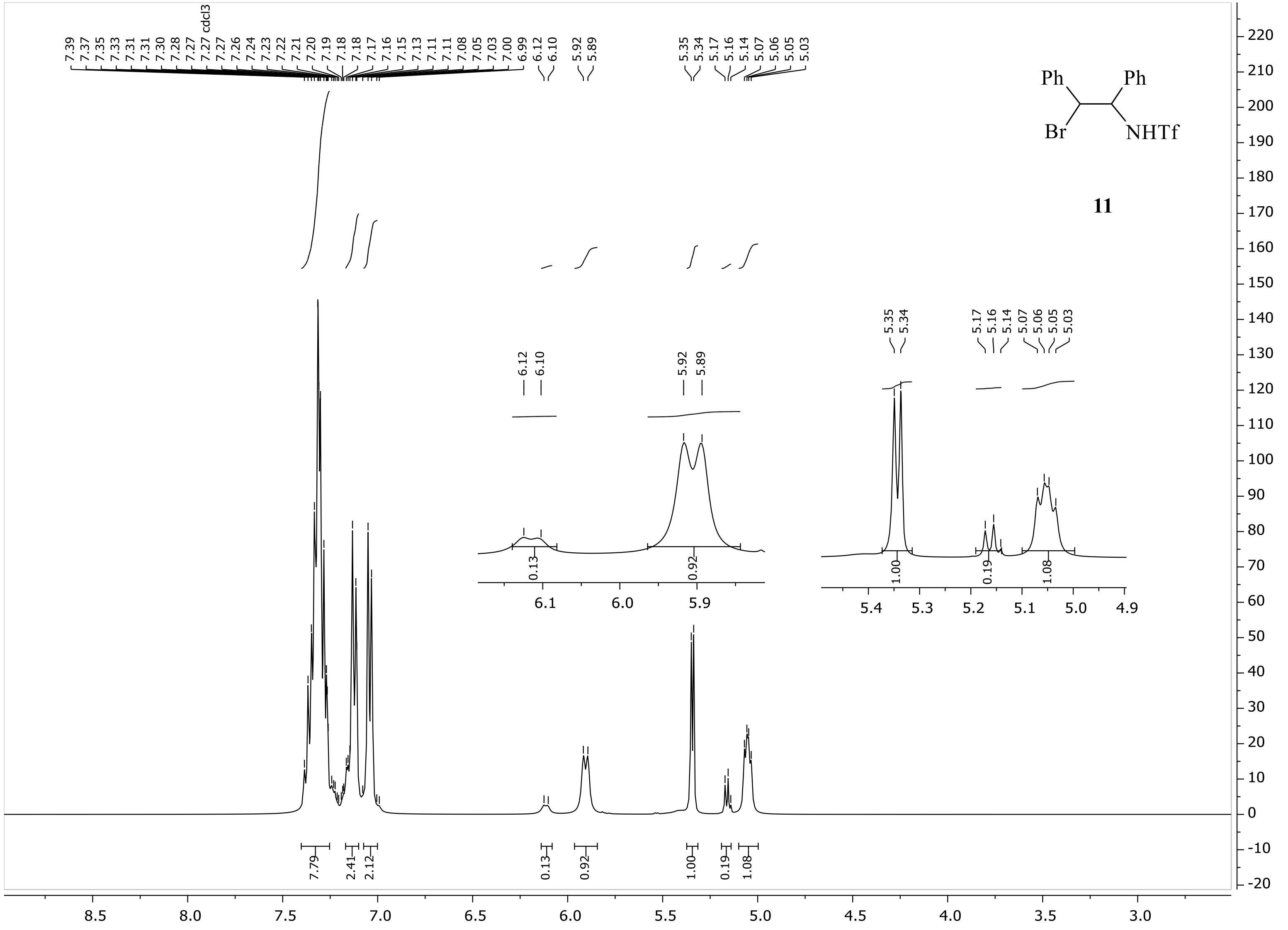

Figure S19. ^{13}C NMR (100.6 MHz, $CDCl_3$) of compound **11**

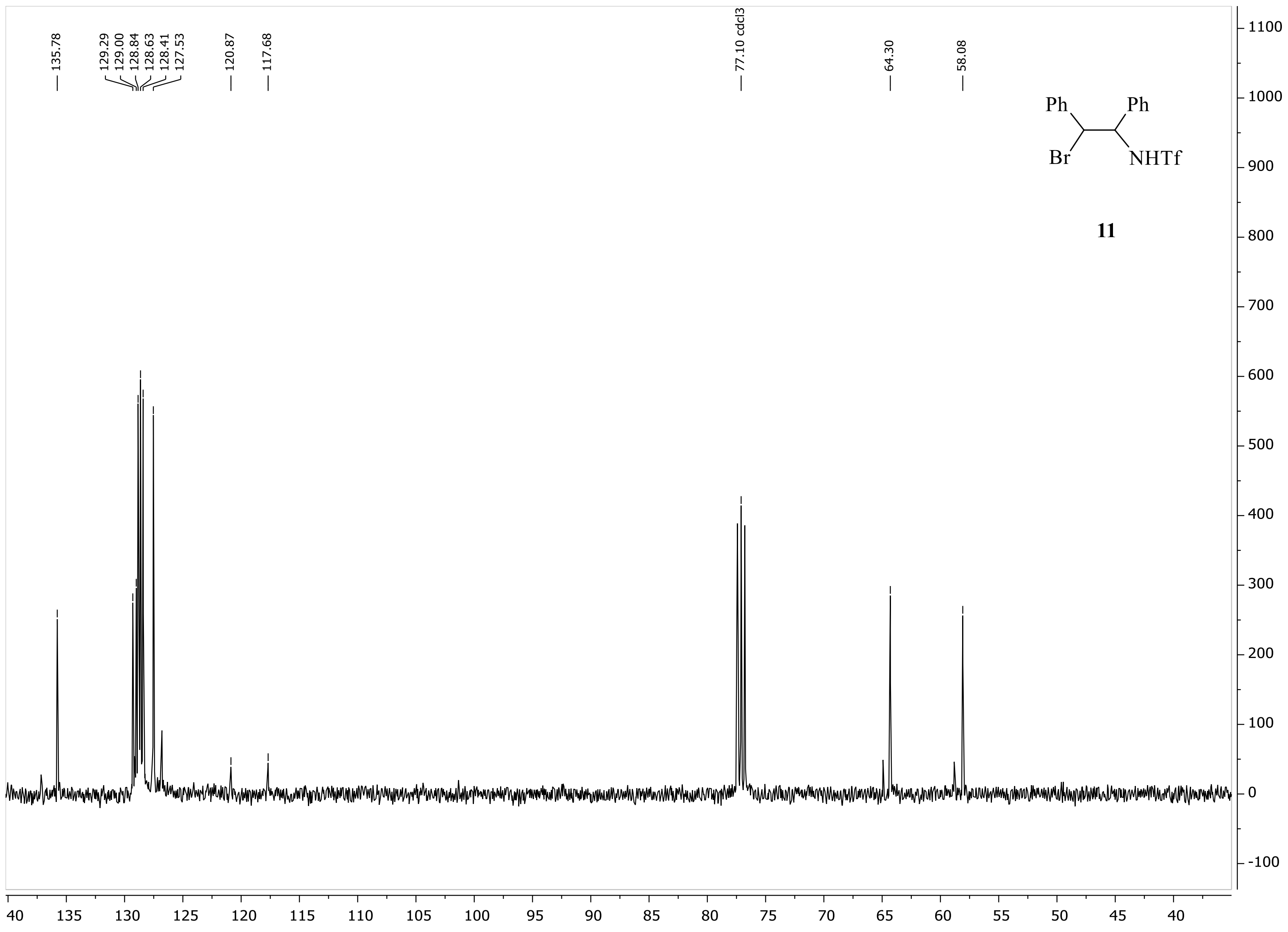

Figure S20. ^{19}F NMR (376 MHz) of compound **11**

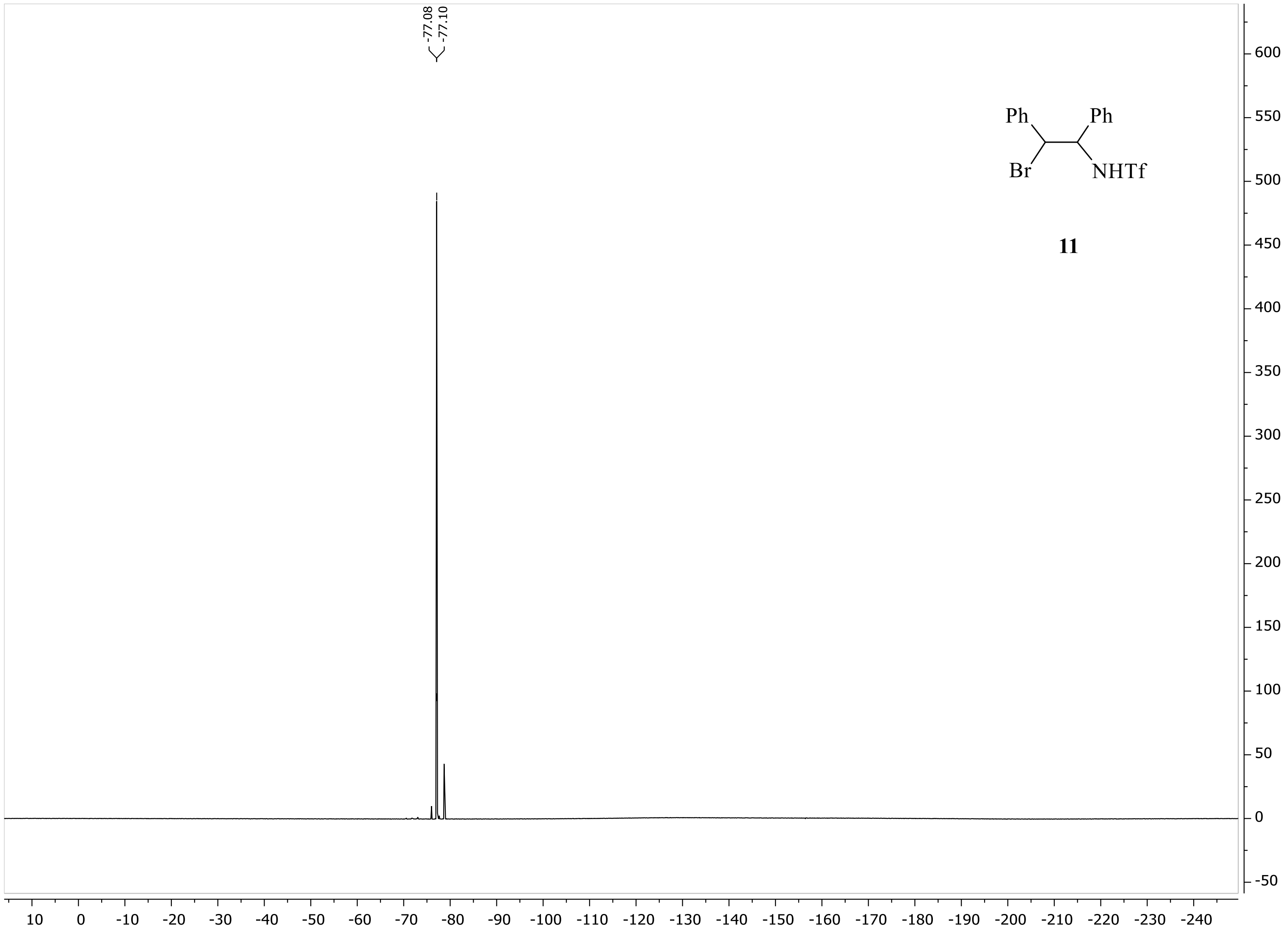

Figure S21. ^{1}H NMR (400.1 MHz, $CDCl_3$) of compounds **13** and **14**

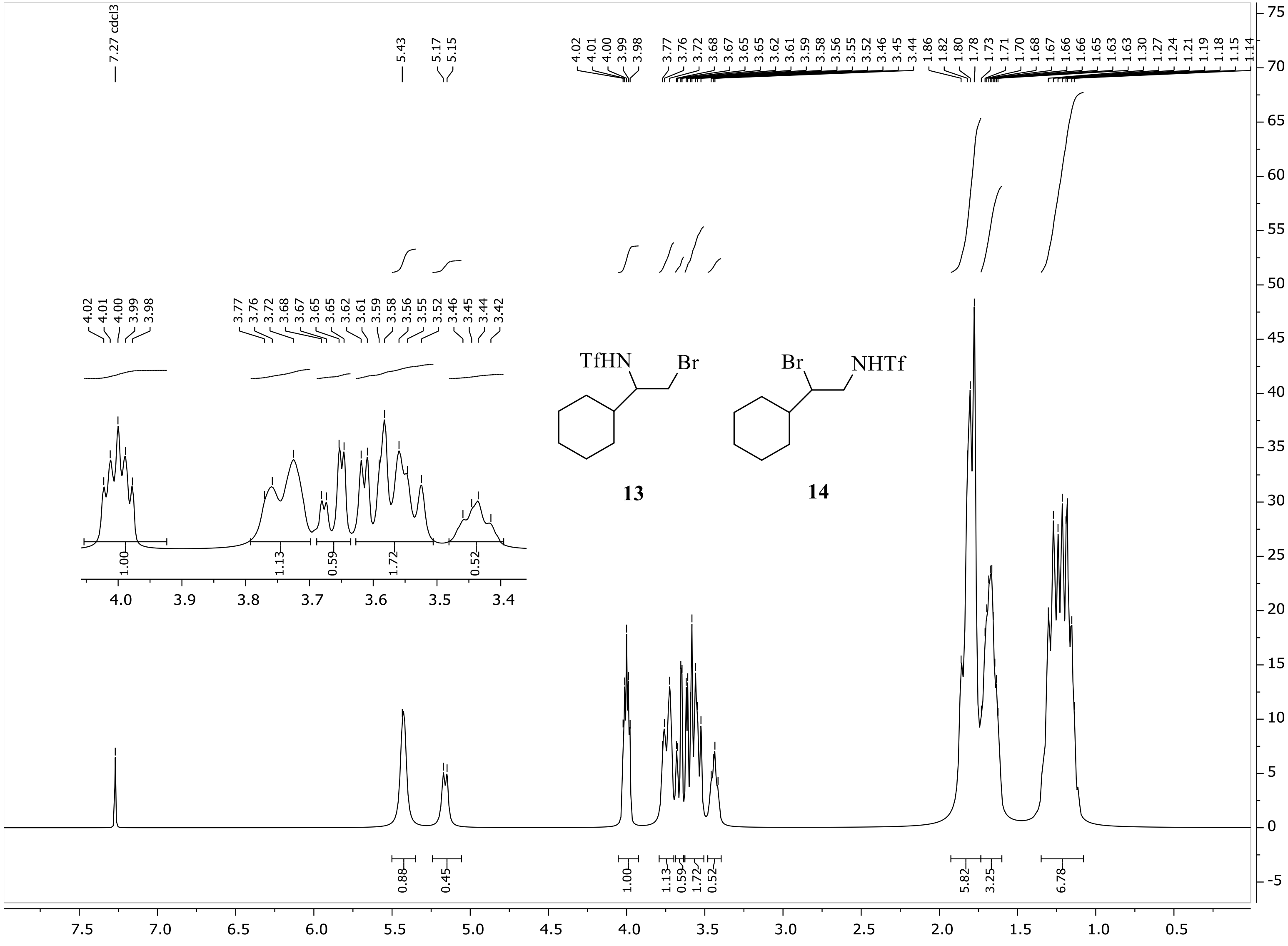

Figure S22. ^{13}C NMR (100.6 MHz, $CDCl_3$) of compounds **13** and **14**

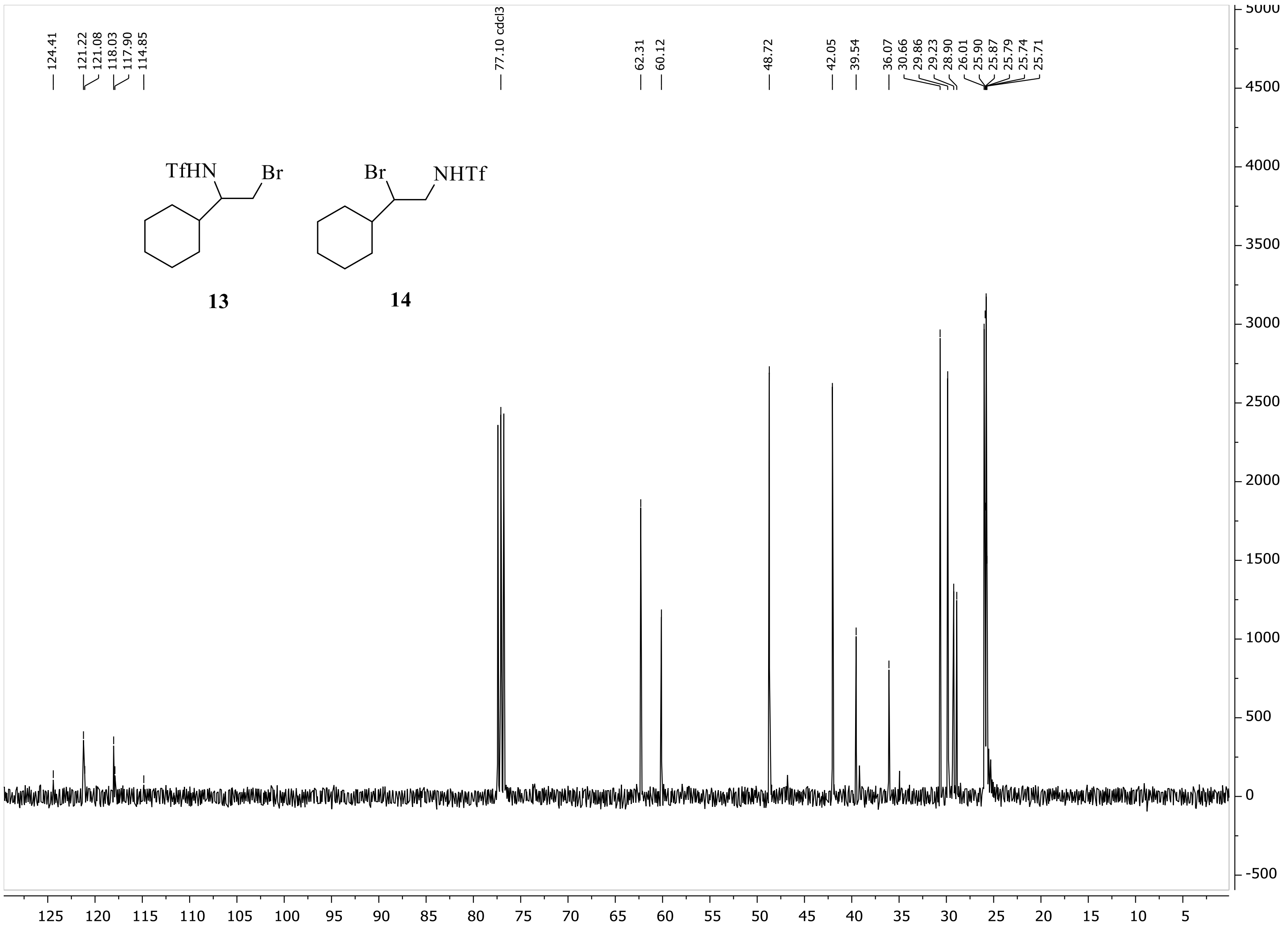

Figure S23. ^{19}F NMR (376 MHz) of compounds **13** and **14**

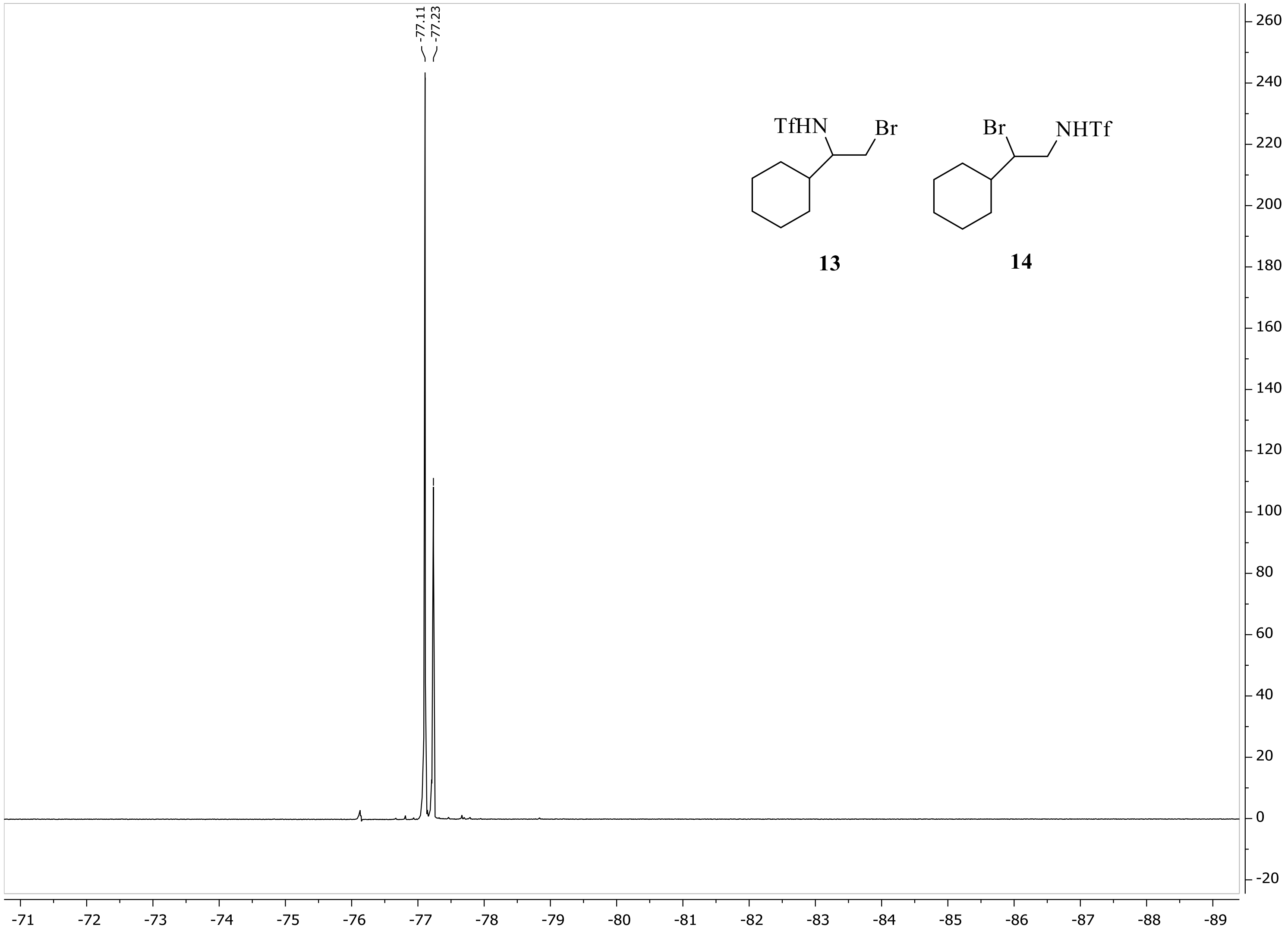

Figure S24. ^{1}H NMR (400.1 MHz, $CDCl_3$) of compound **14**

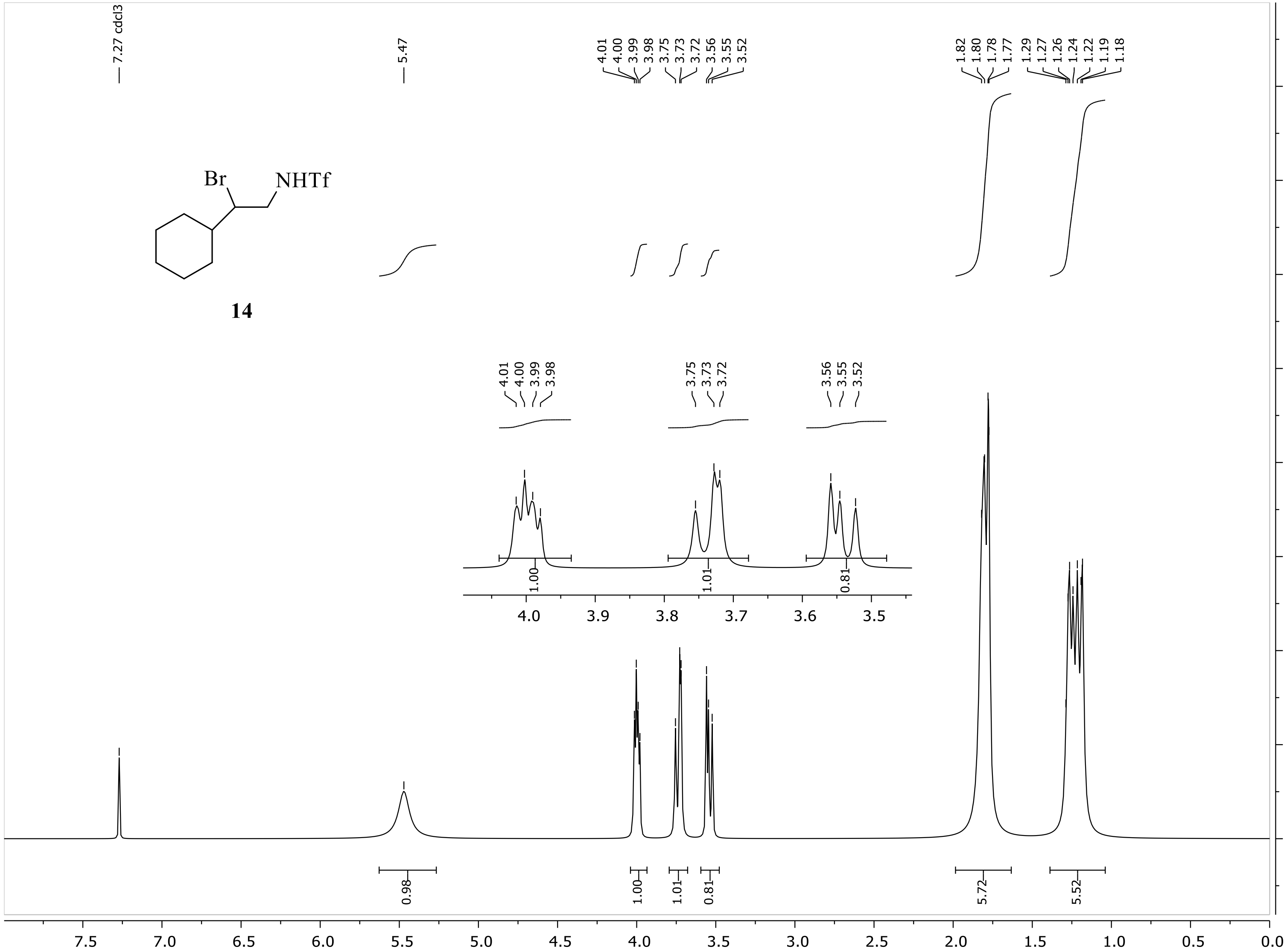

Figure S25. ^{13}C NMR (100.6 MHz, $CDCl_3$) of compound **14**

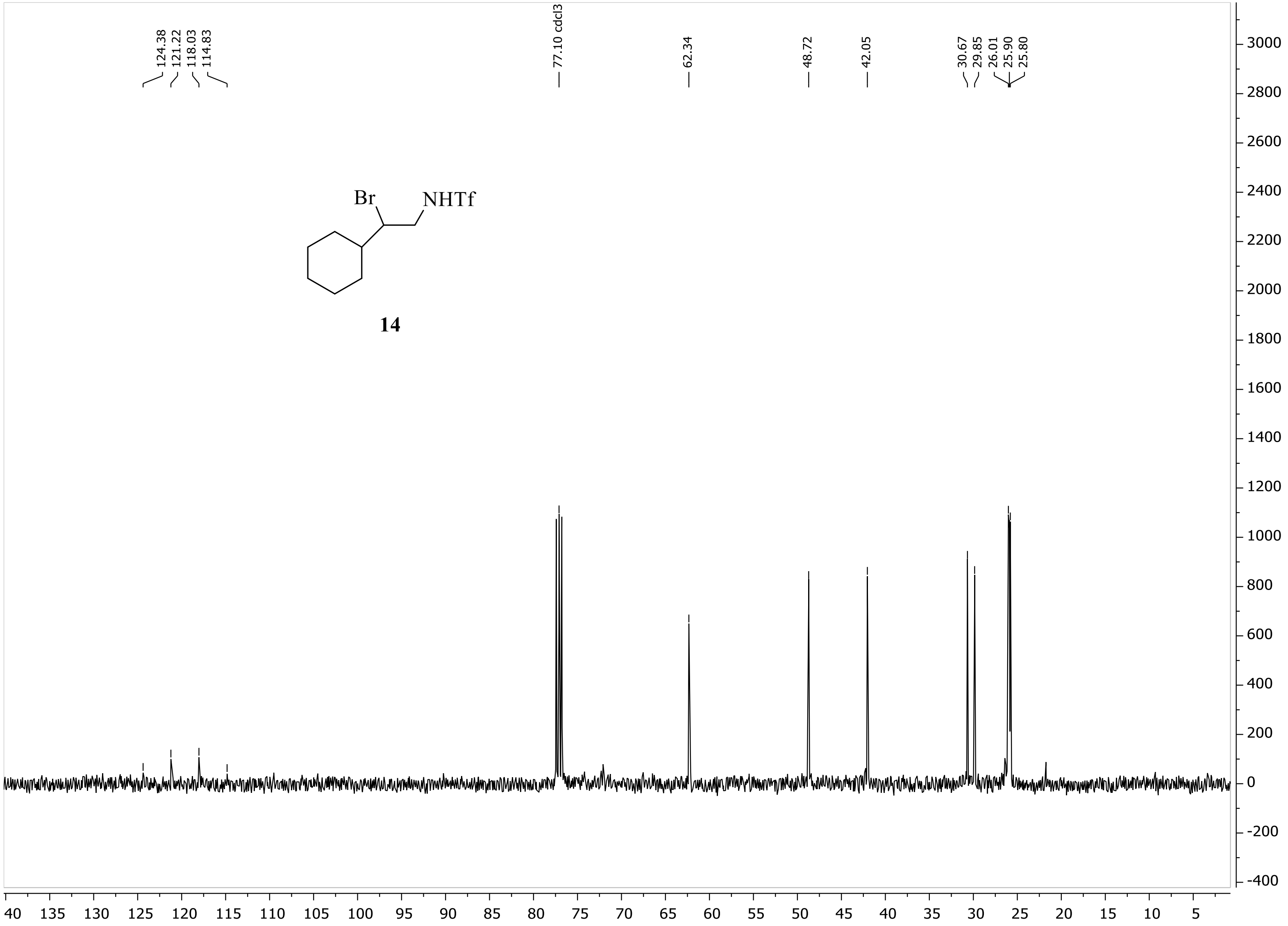

Figure S26. ^{1}H NMR (400.1 MHz, $CDCl_3$) of compounds **15** and **16**

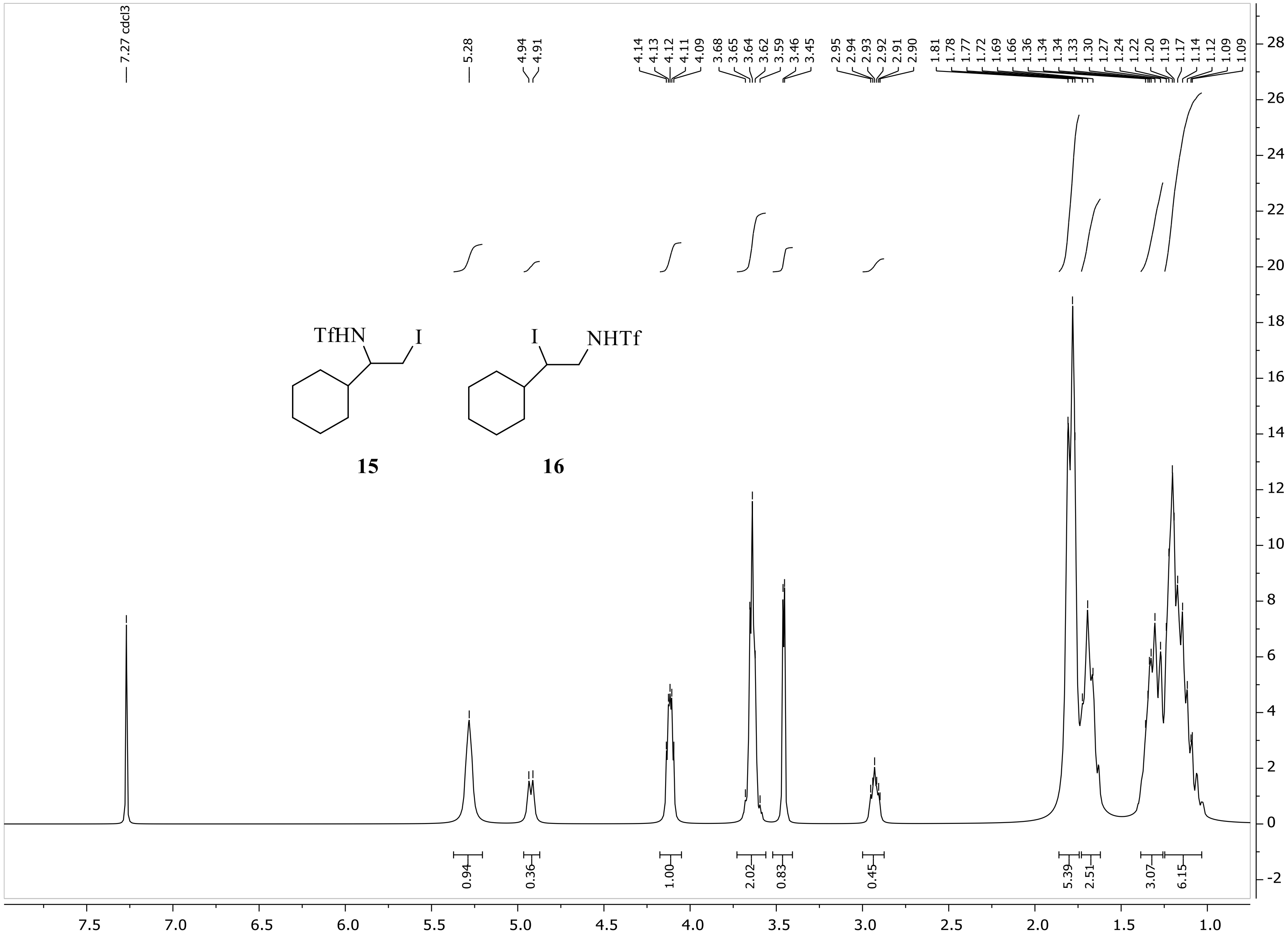

Figure S27. ^{13}C NMR (100.6 MHz, $CDCl_3$) of compounds **15** and **16**

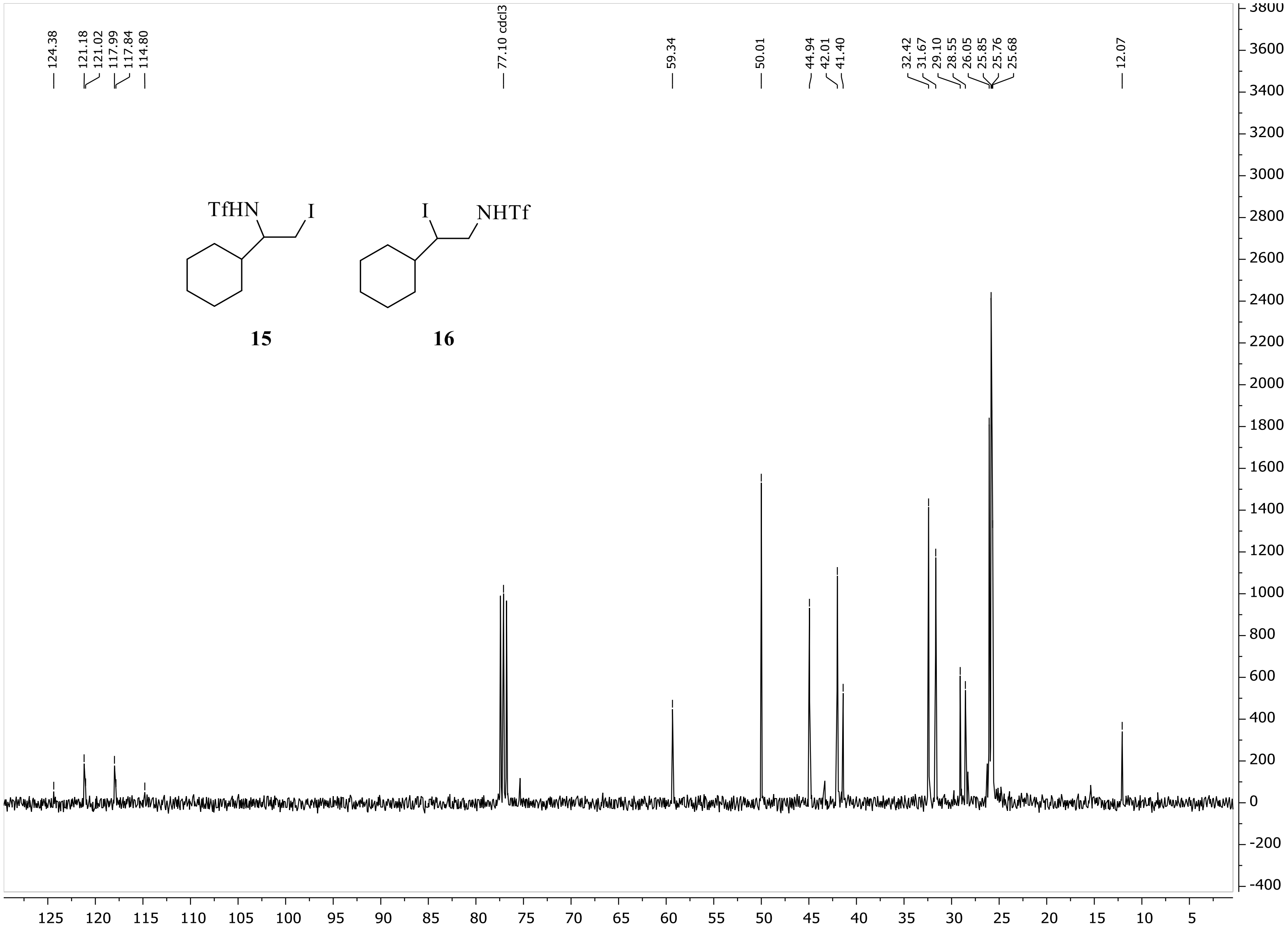

Figure S28. ^{19}F NMR (376 MHz) of compounds **15** and **16**

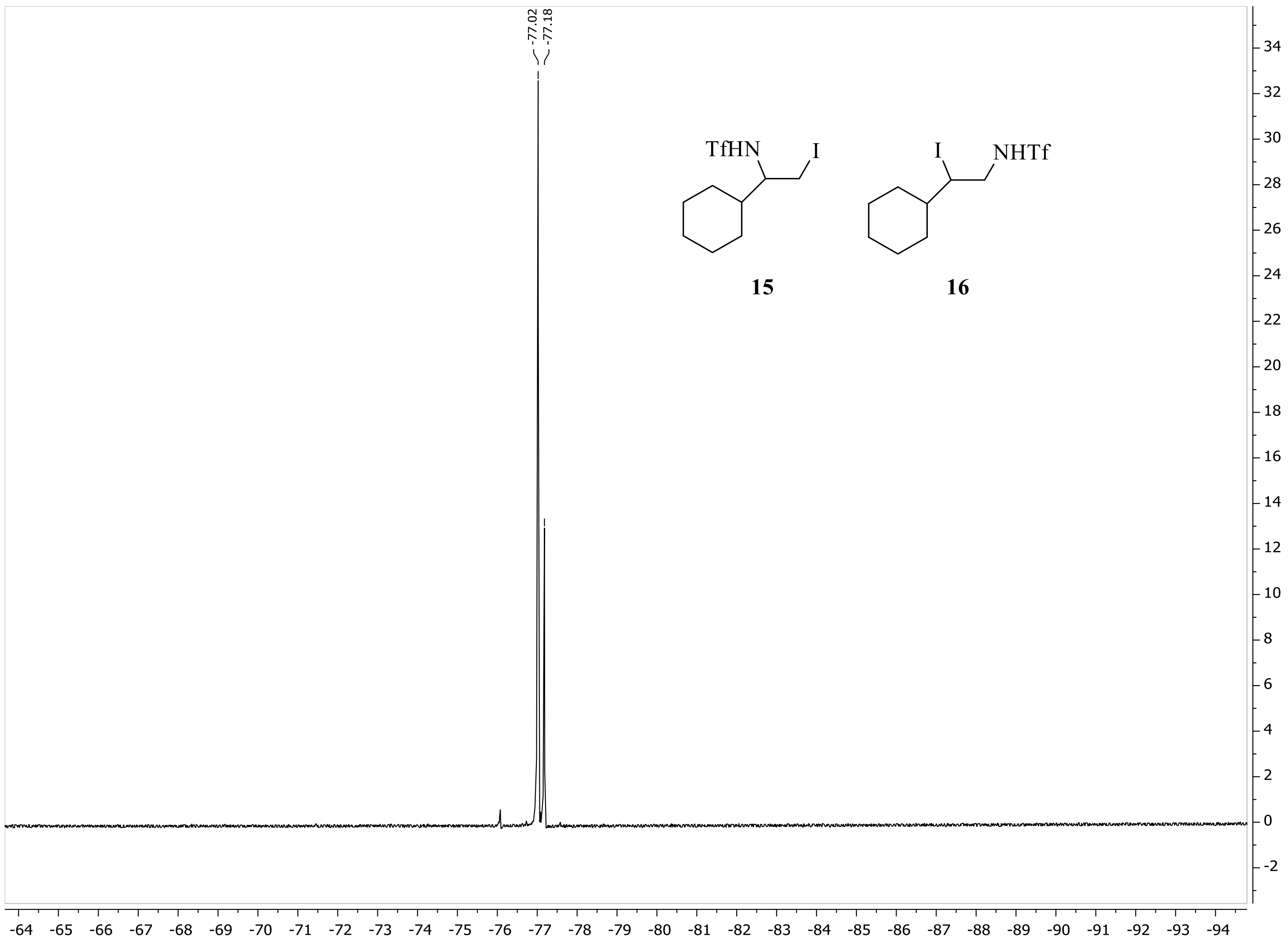

Figure S29. ¹H NMR (400.1 MHz, $CDCl_3$) of compounds **17** and **18**

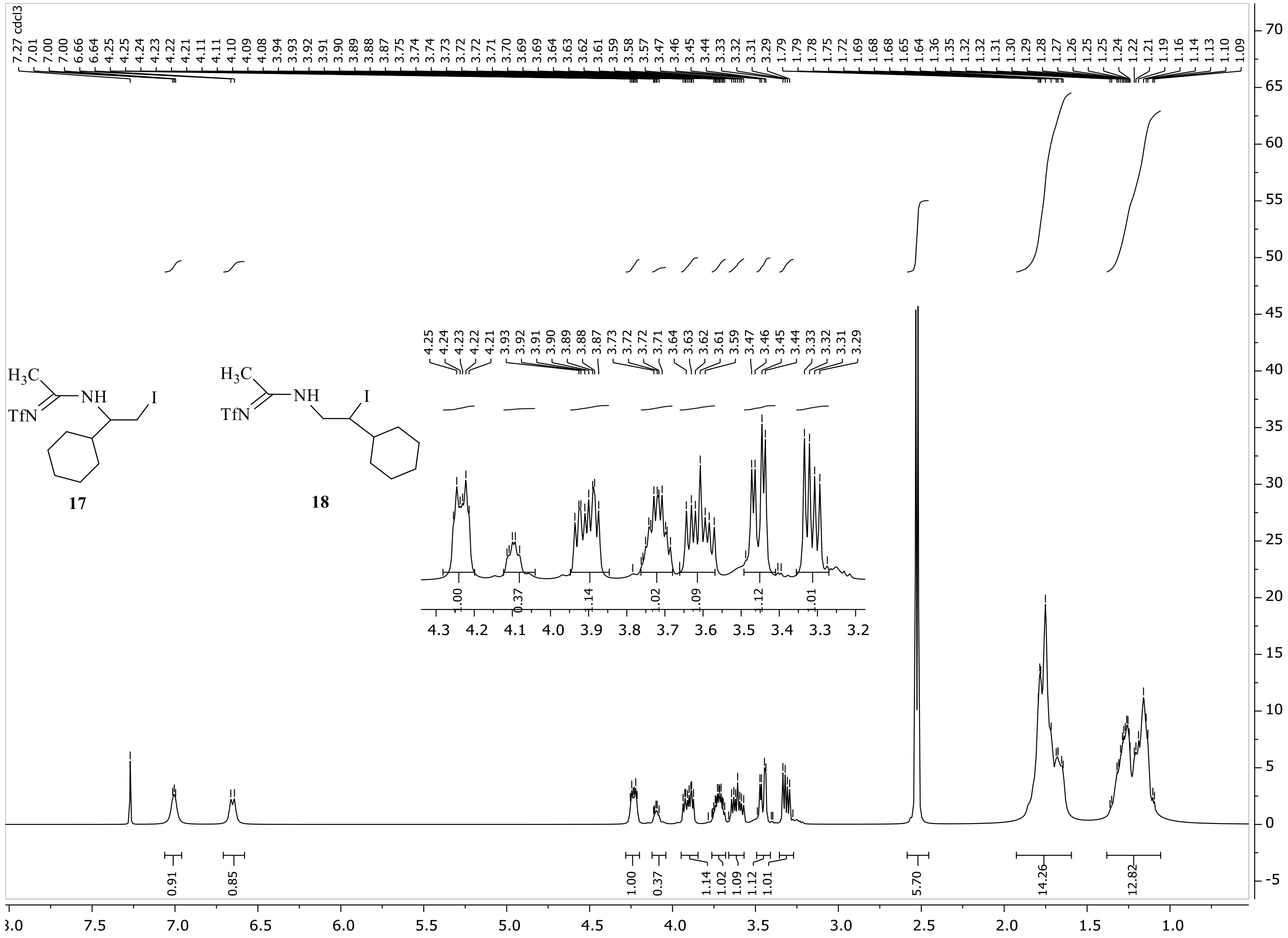

Figure S30. ^{13}C NMR (100.6 MHz, $CDCl_3$) of compounds **17** and **18**

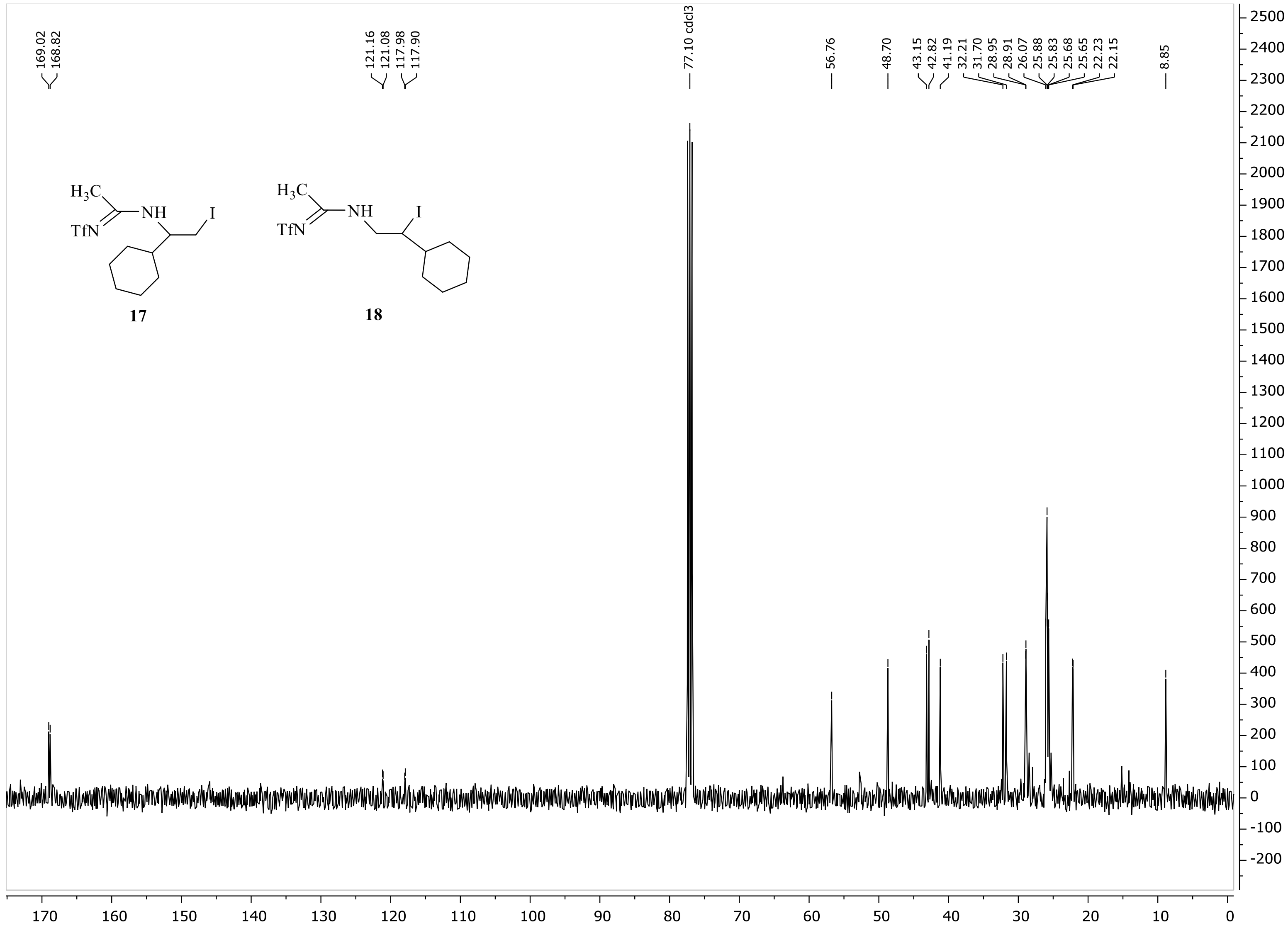

Figure S31. ^{13}C J-modulation NMR (100.6 MHz, $CDCl_3$) spectrum of **17** and **18**

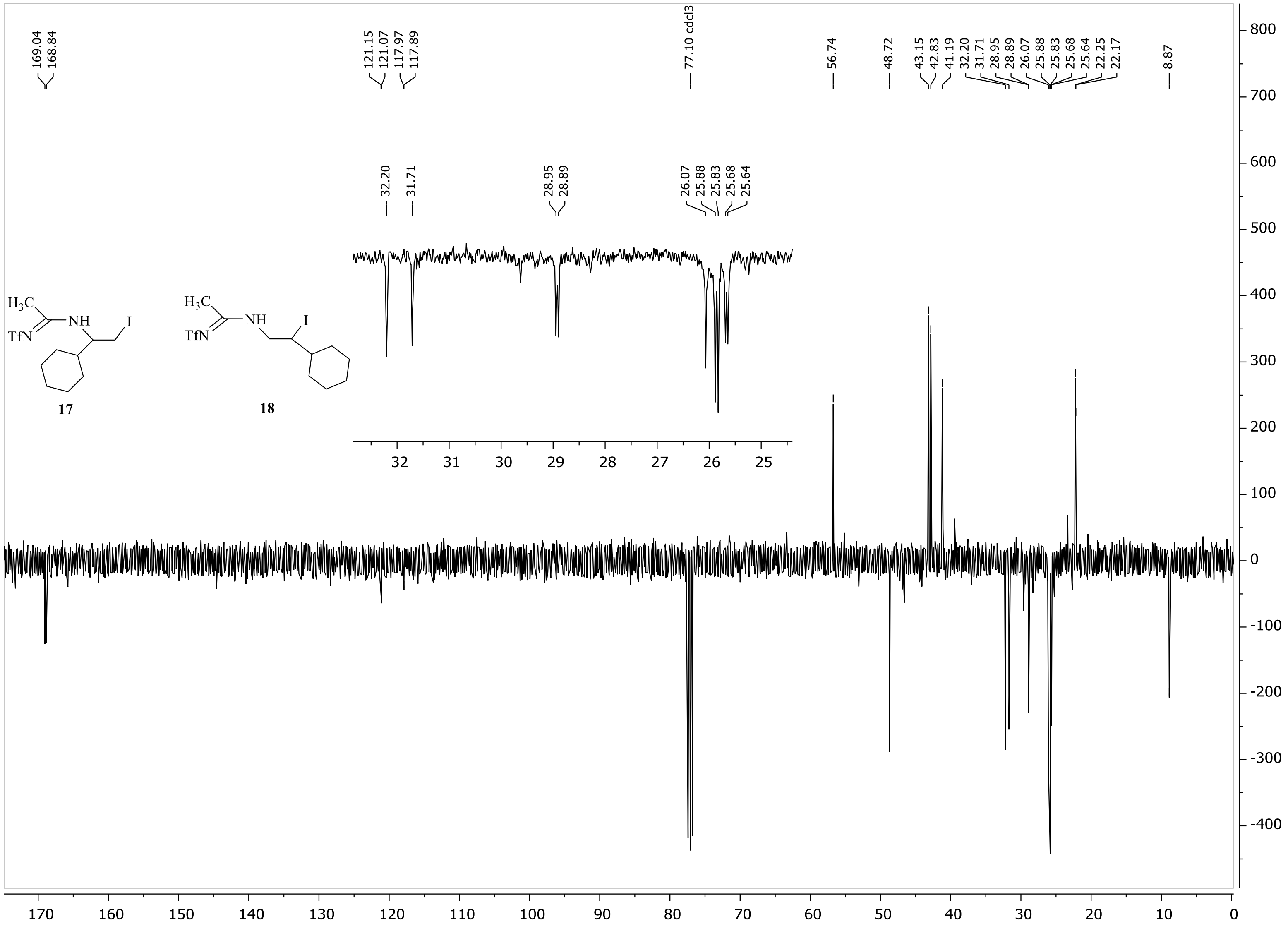

Figure S32. ^{19}F NMR (376 MHz) of compounds **17** and **18**

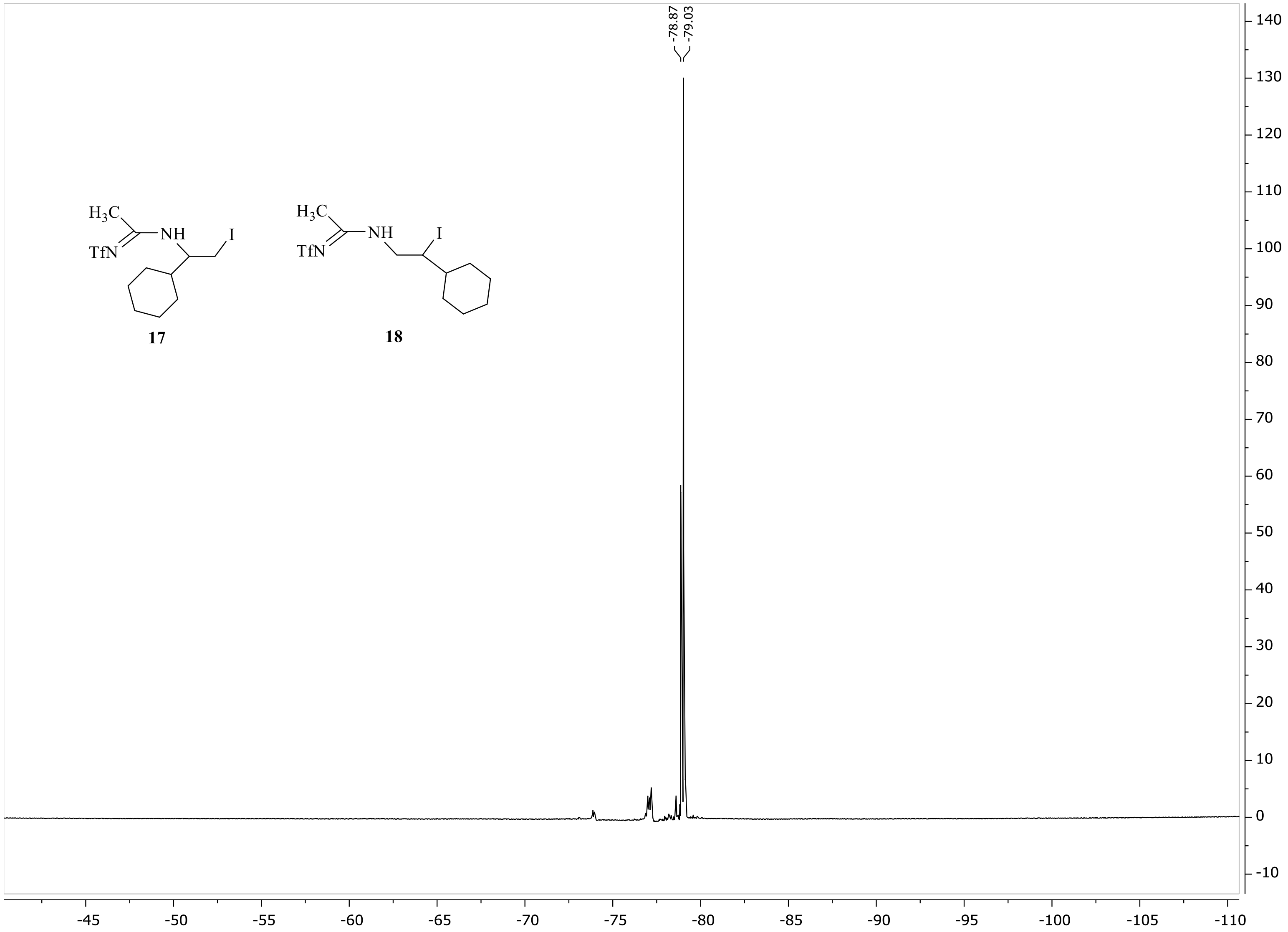

Figure S33. ^{1}H NMR (400.1 MHz, $CDCl_3$) of compound **18**

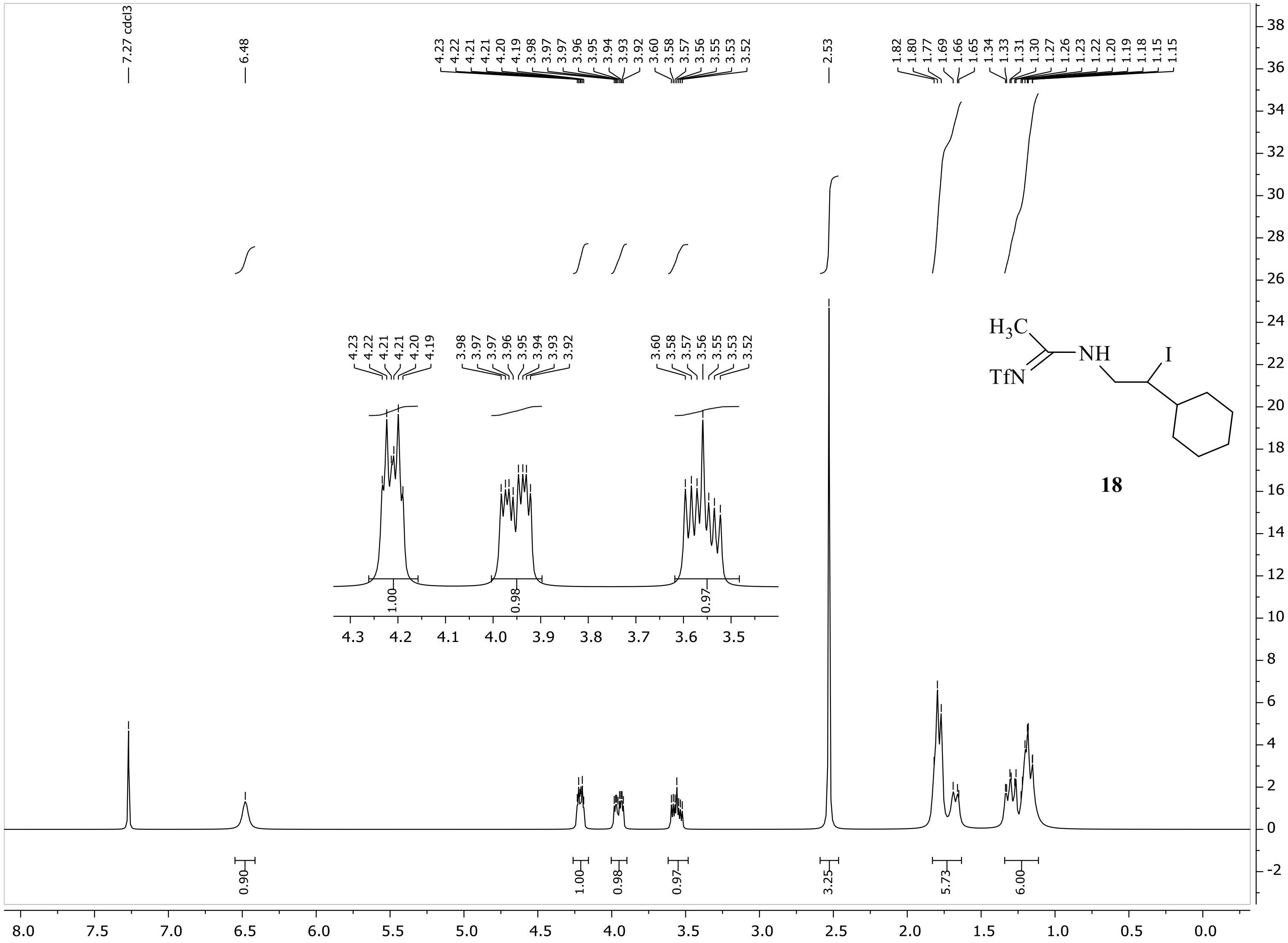

Figure S34. ^{13}C NMR (100.6 MHz, $CDCl_3$) of compound **18**

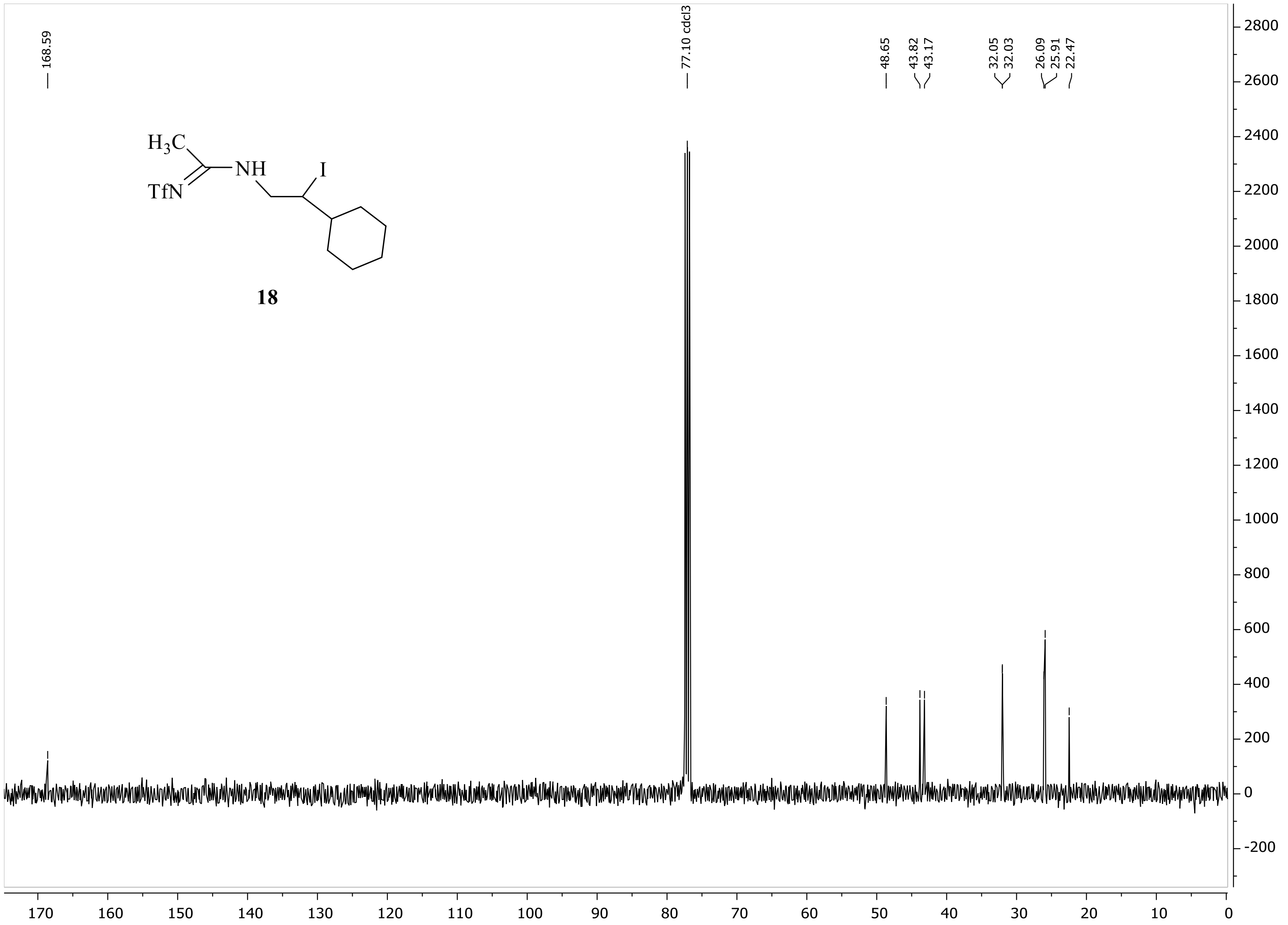

Figure S35. ^{19}F NMR (376 MHz) of compound **18**

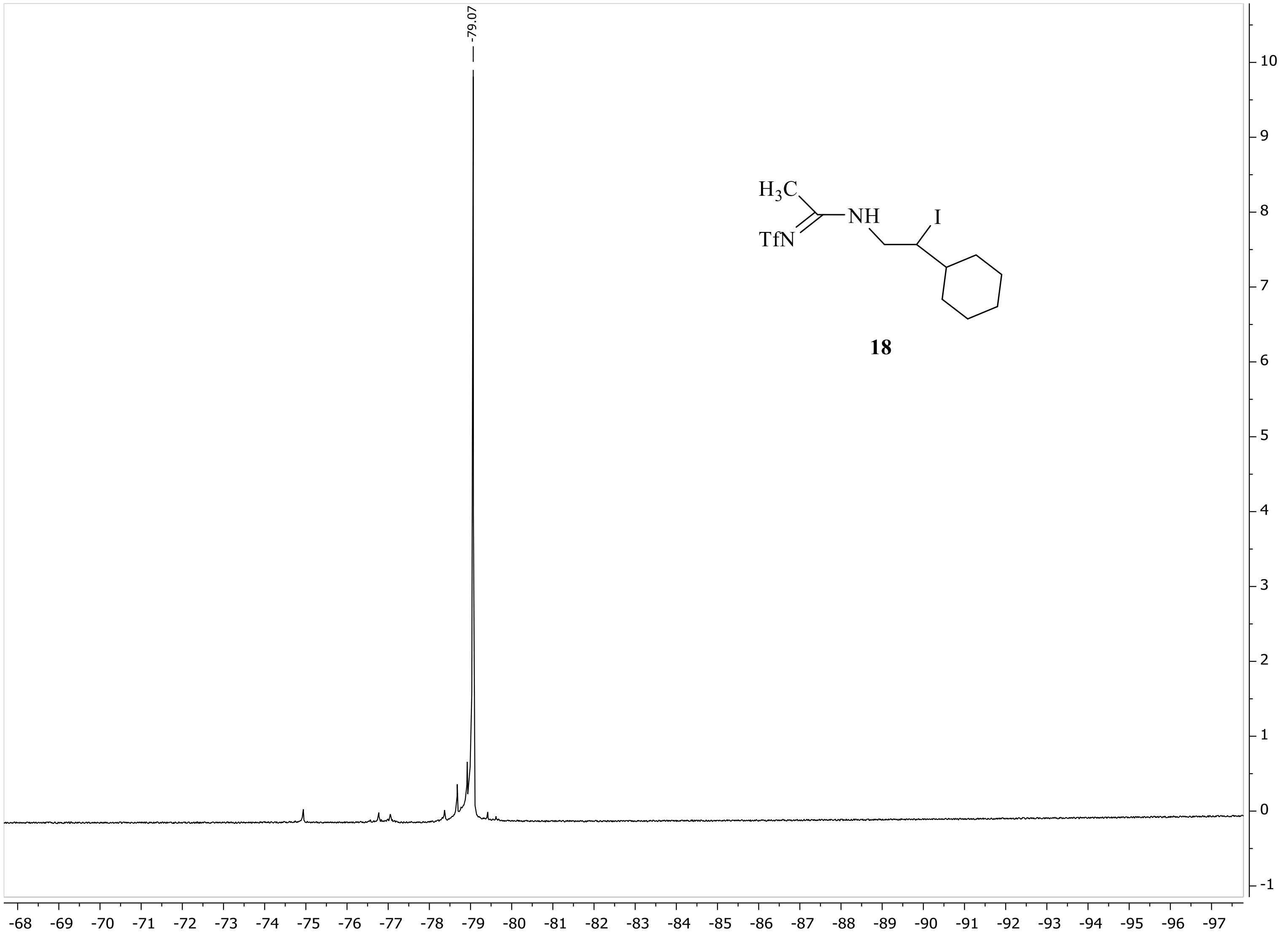

Figure S36. ^{1}H NMR (400.1 MHz, $CDCl_3$) of compound **19**

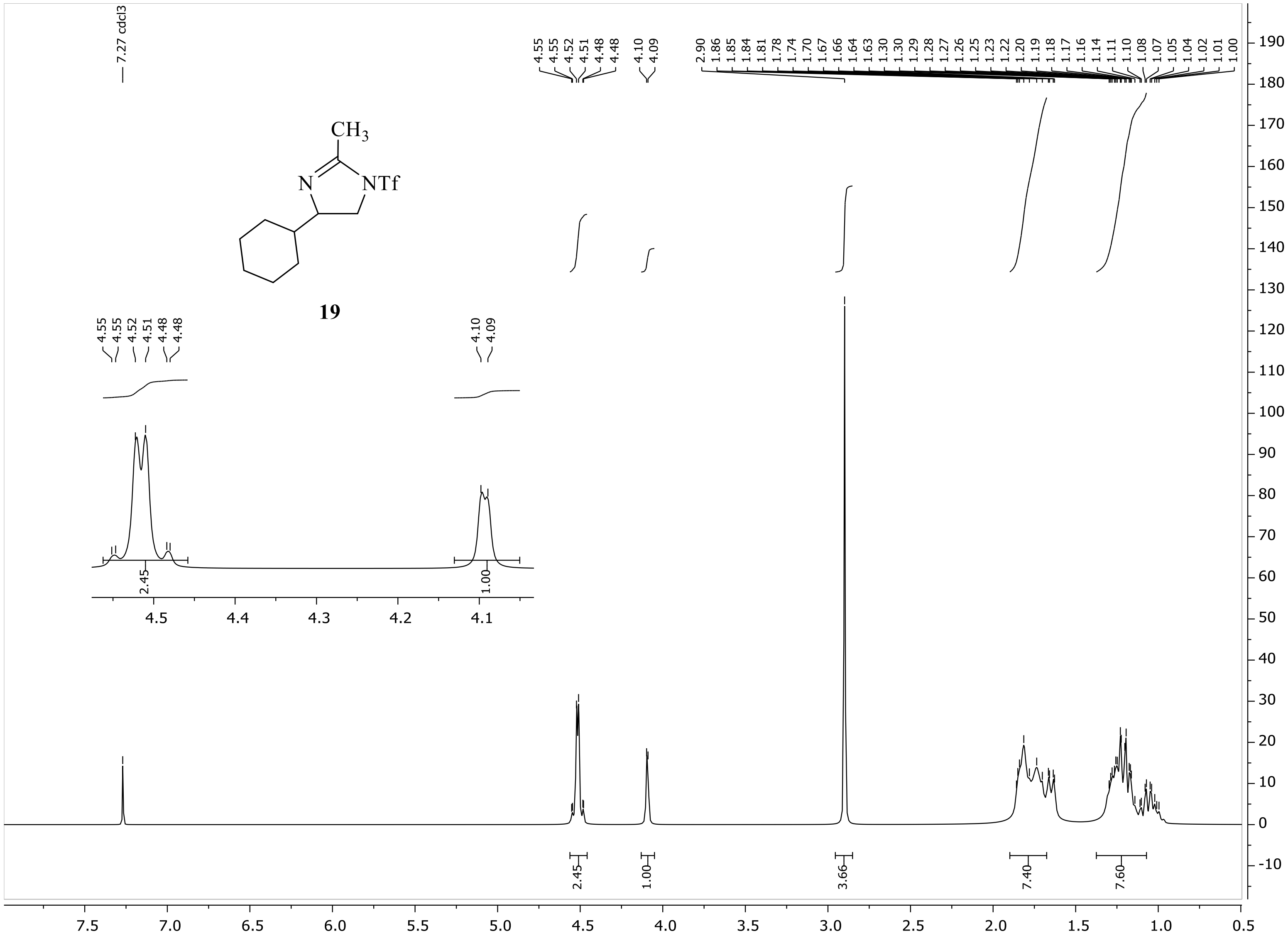

Figure S37. ^{13}C NMR (100.6 MHz, $CDCl_3$) of compound **19**

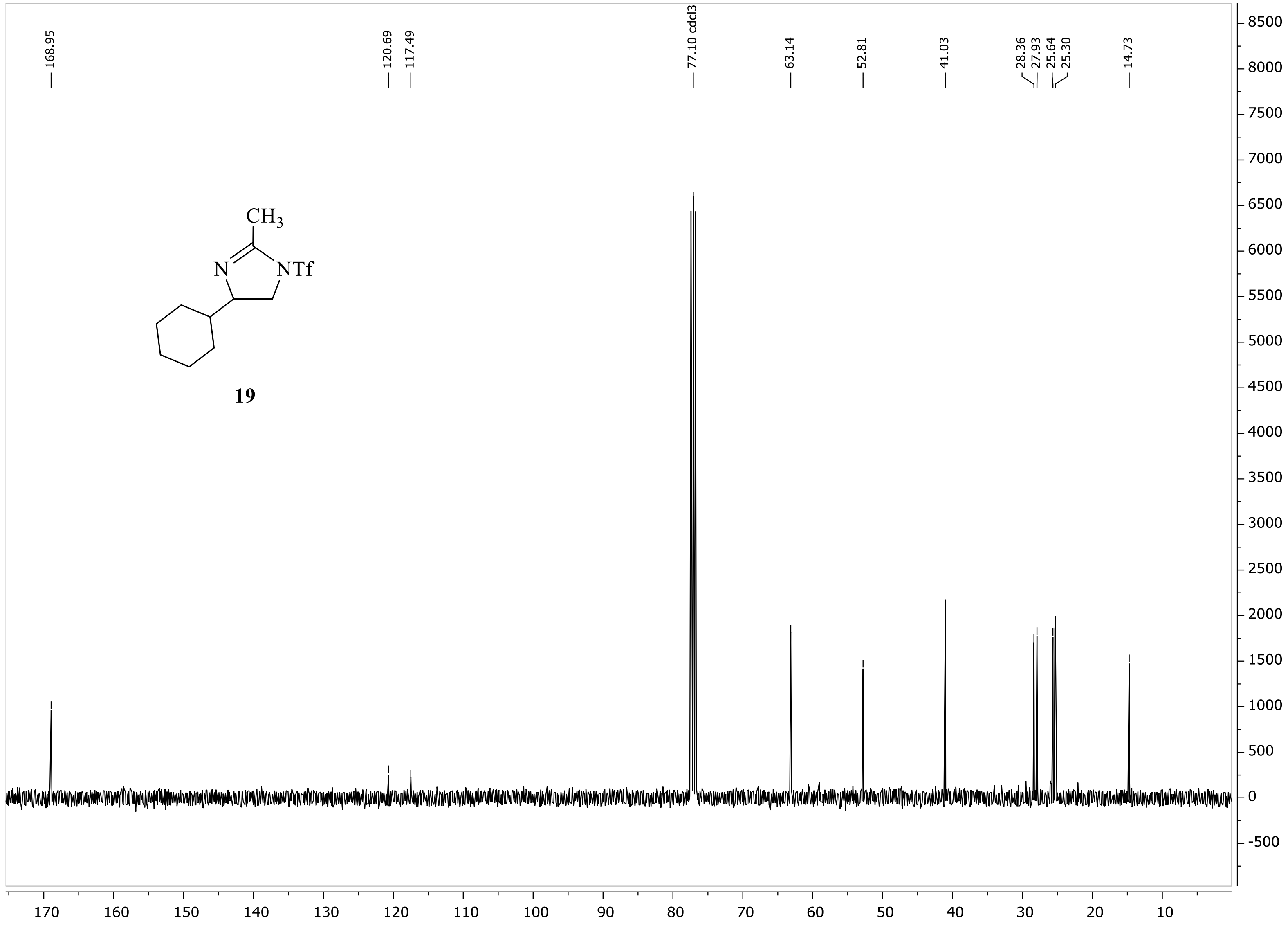

Figure S38. ^{13}C J-modulation NMR (100.6 MHz, CDCl3) of compound **19**

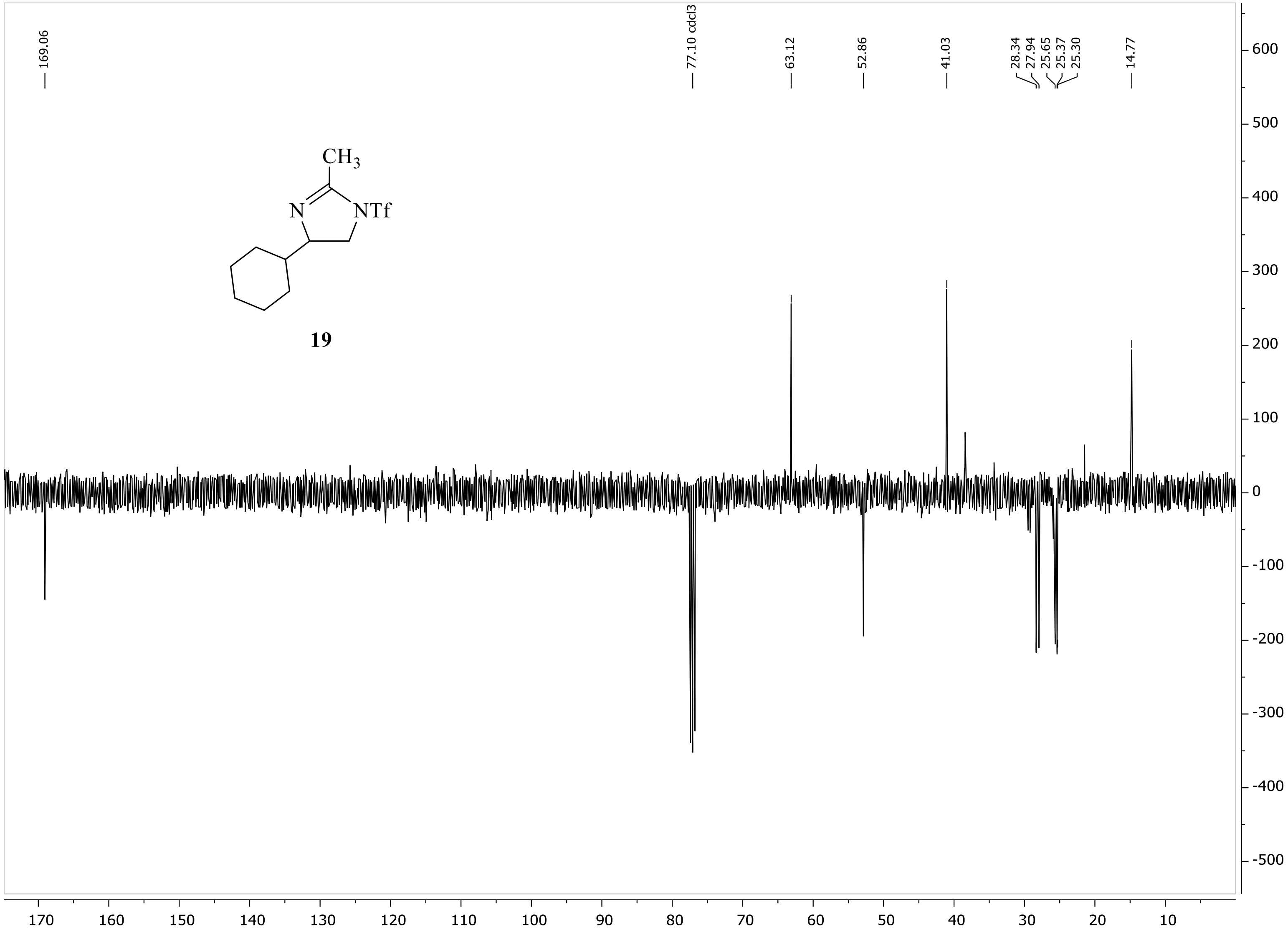

Figure S39. ^{19}F NMR (376 MHz) of compound **19**

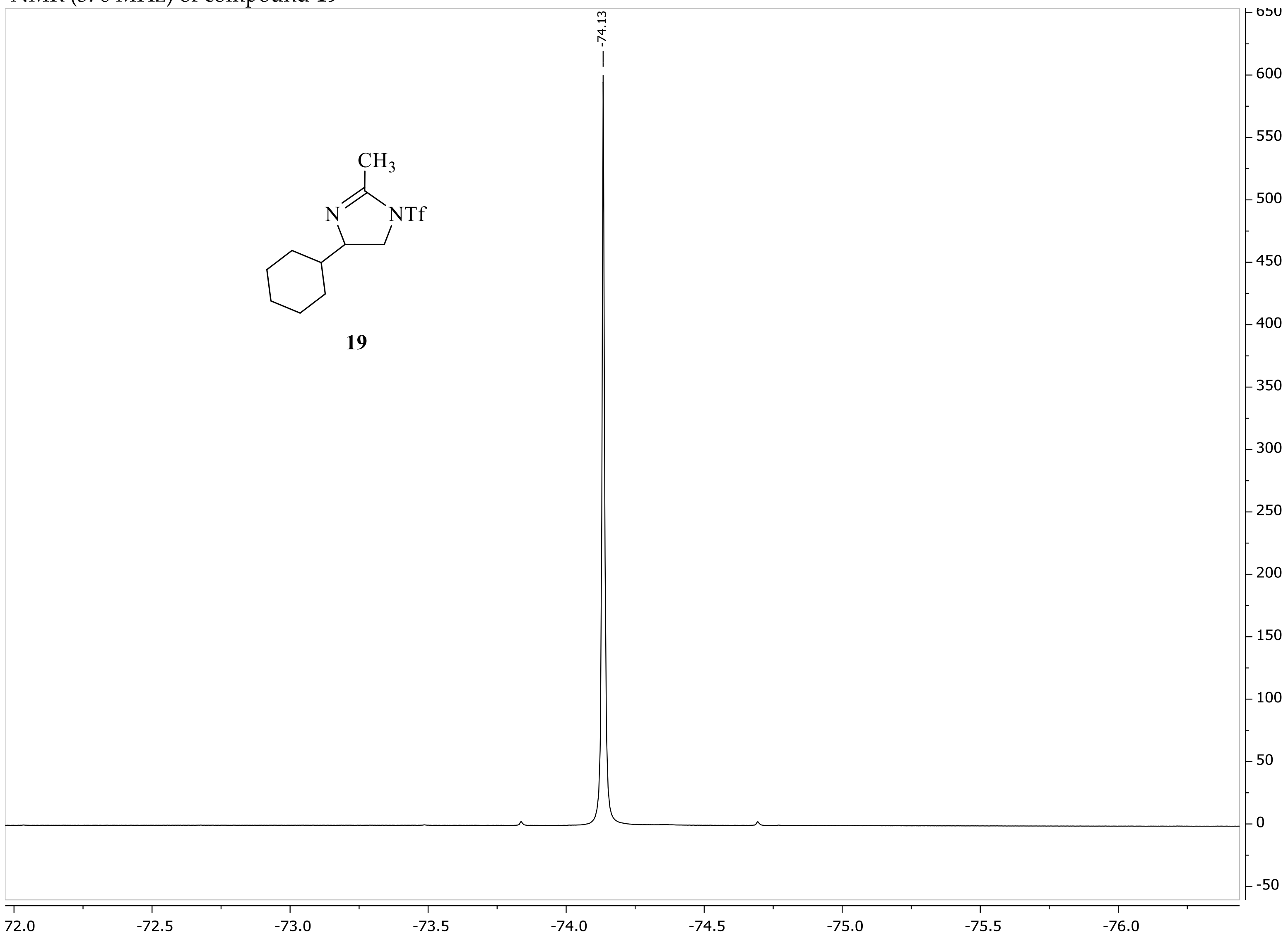

1.2 Copies of HRMS spectra

Figure S40. HRMS of compound **9**

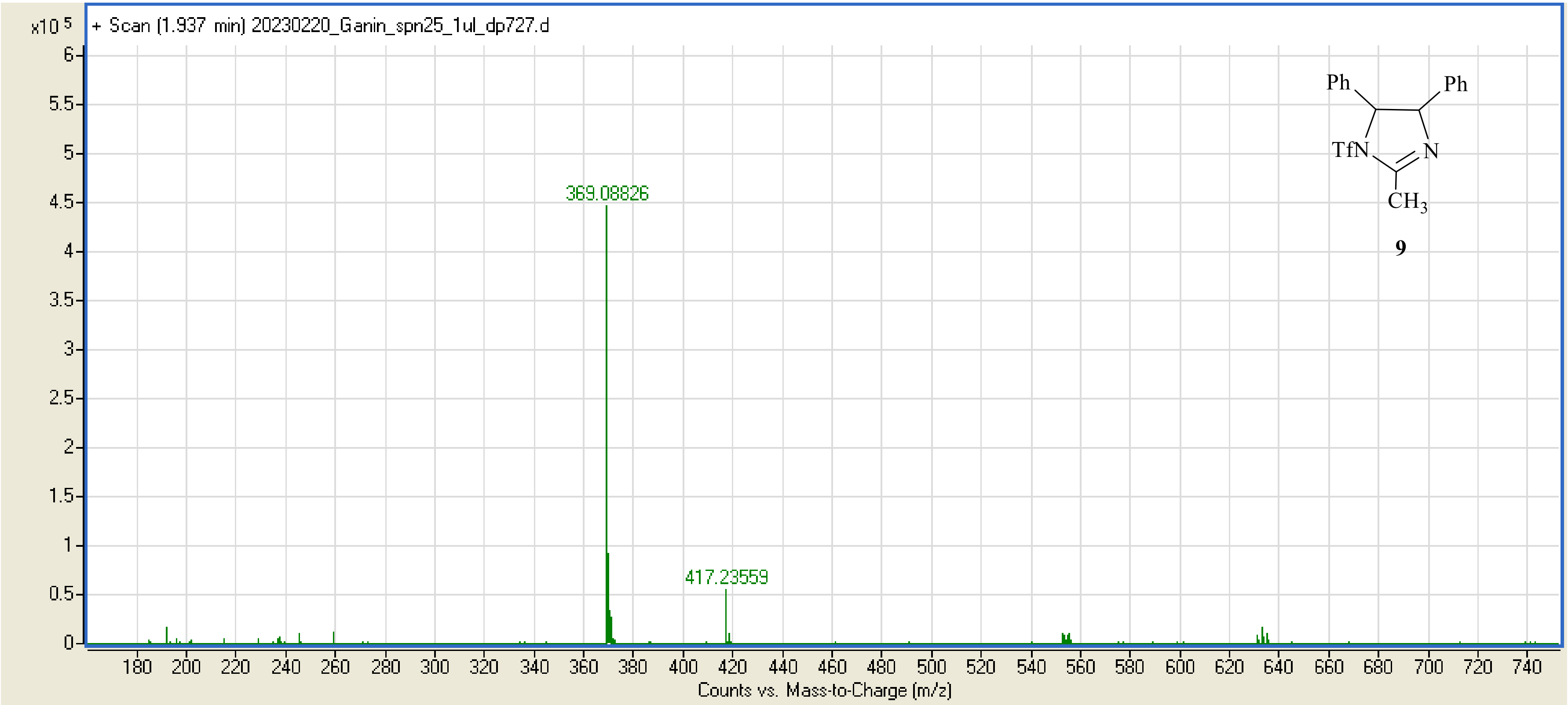

Figure S41. HRMS of compound **10**

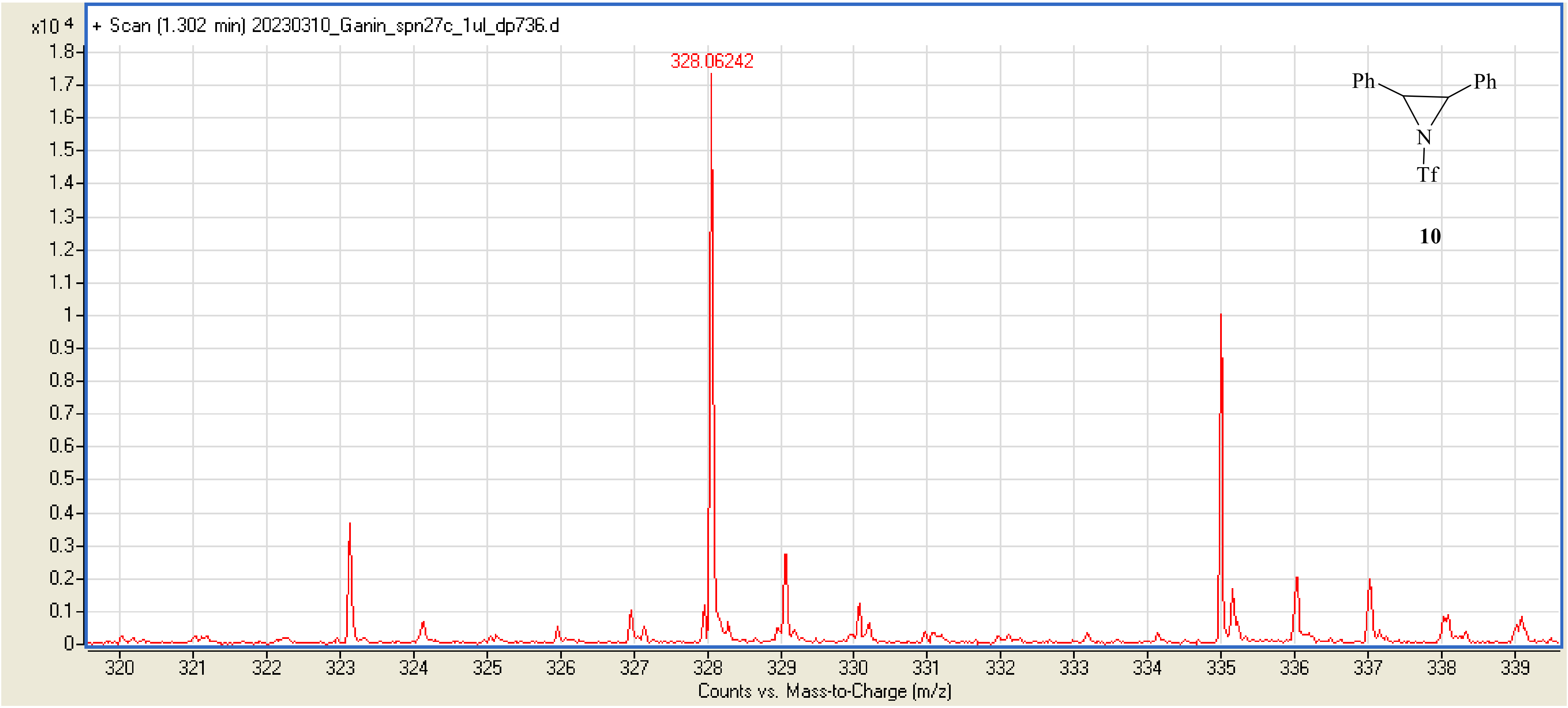

Figure S42. HRMS of compound **11**

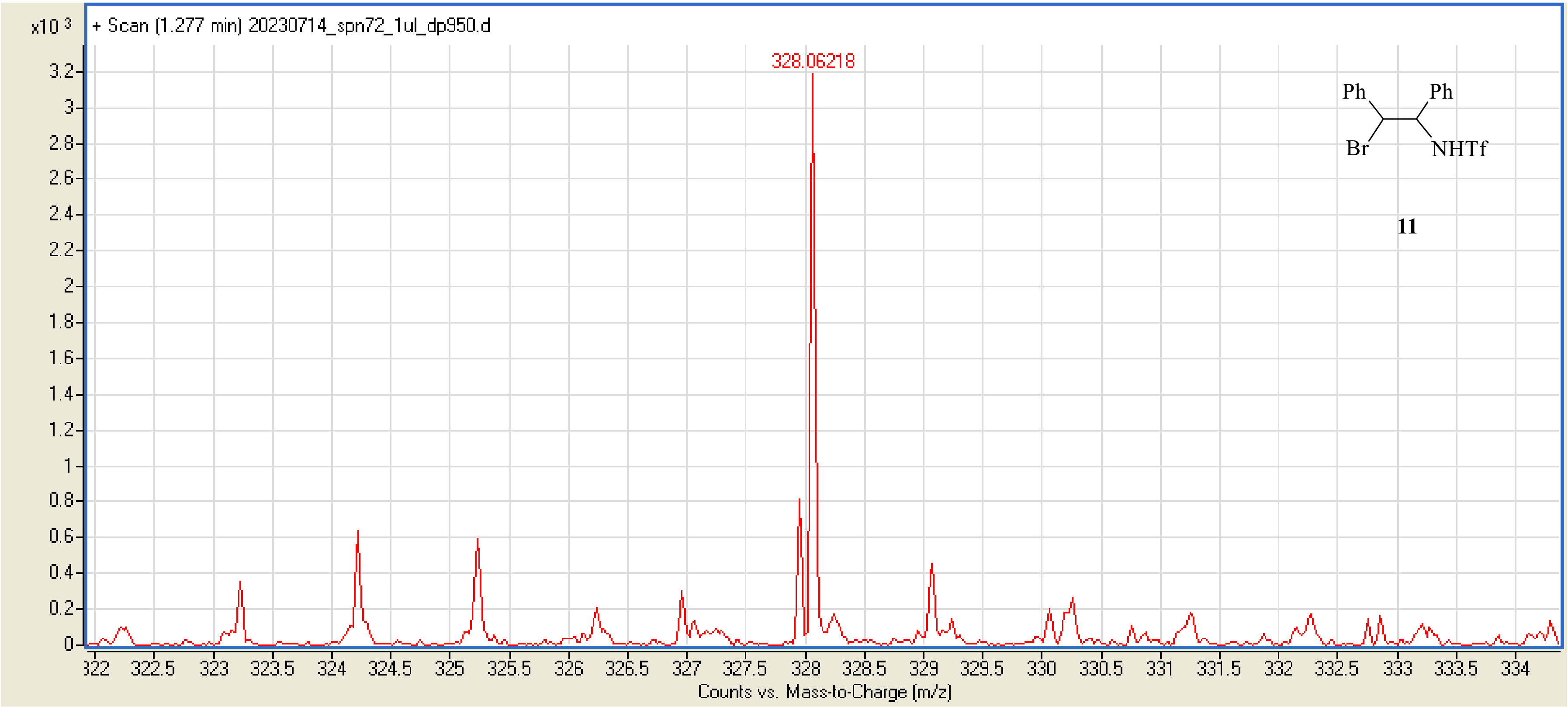

Figure S43. HRMS of compound **18**

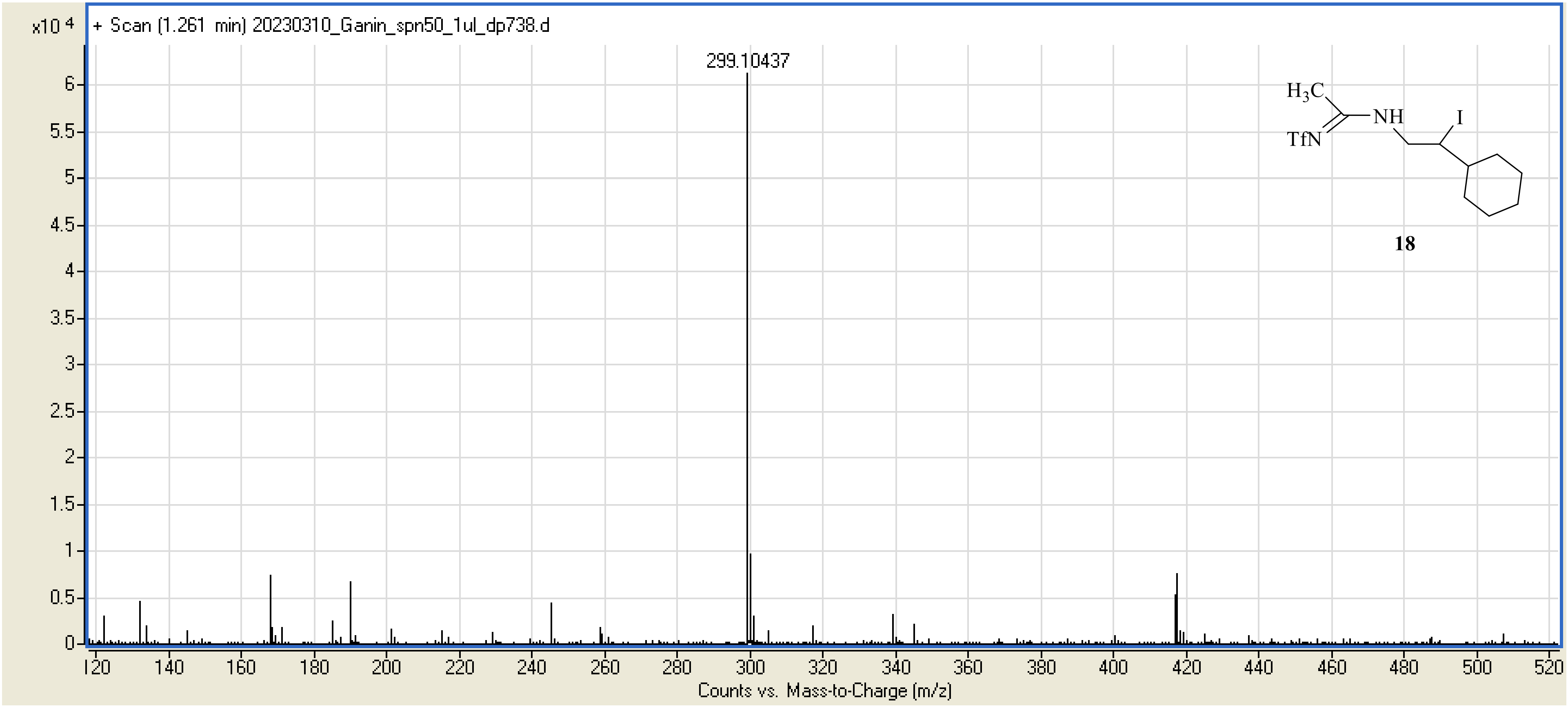

Figure S44. HRMS of compound **19**

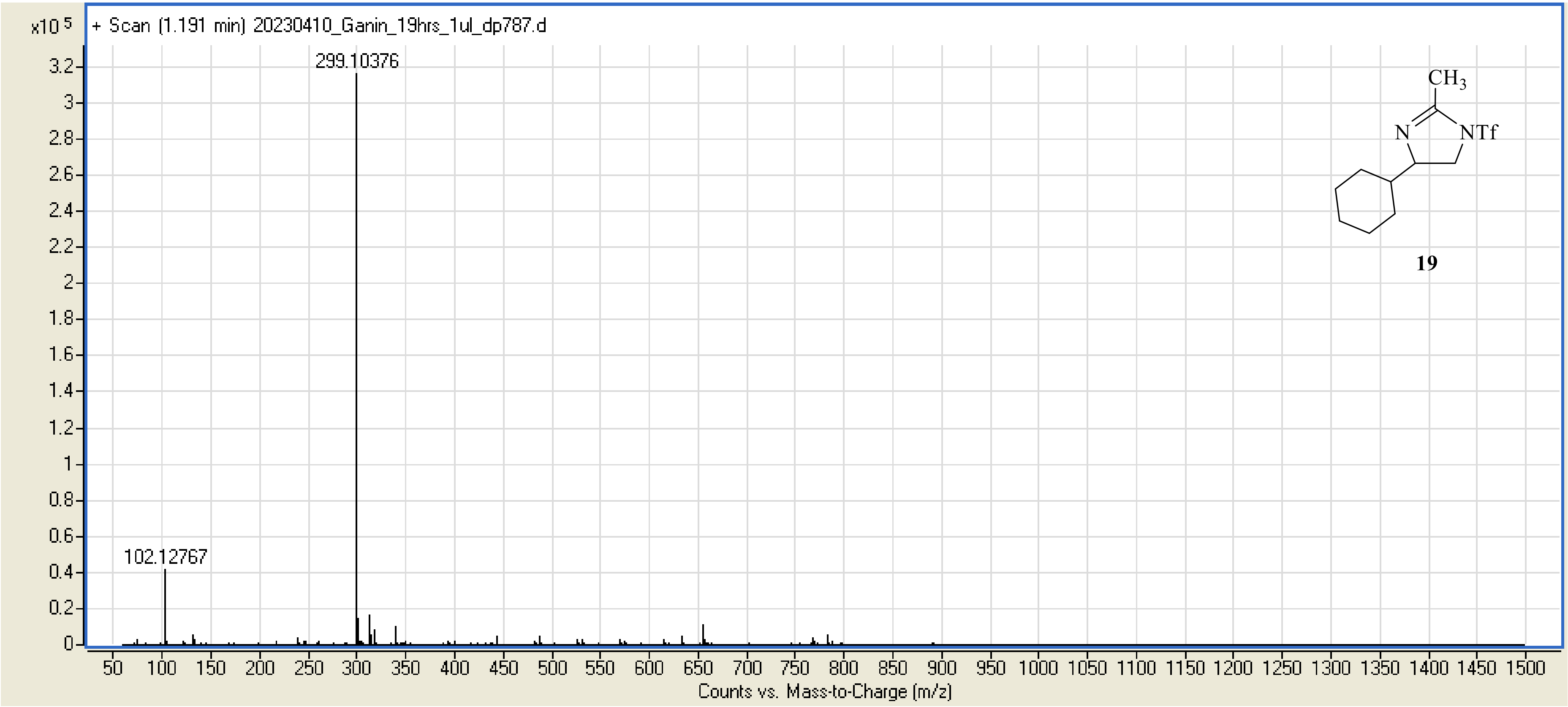

Supplement: Supplementary file 1 [file ijms-24-15947-s001.zip › ijms-2675362-supplementary.pdf]
